# Nuclear and mitochondrial genetic variants associated with mitochondrial DNA copy number

**DOI:** 10.1038/s41598-024-52373-0

**Published:** 2024-01-24

**Authors:** Adriana Koller, Michele Filosi, Hansi Weissensteiner, Federica Fazzini, Mathias Gorski, Cristian Pattaro, Sebastian Schönherr, Lukas Forer, Janina M. Herold, Klaus J. Stark, Patricia Döttelmayer, Andrew A. Hicks, Peter P. Pramstaller, Reinhard Würzner, Kai-Uwe Eckardt, Iris M. Heid, Christian Fuchsberger, Claudia Lamina, Florian Kronenberg

**Affiliations:** 1 Institute of Genetic Epidemiology, Medical University of Innsbruck, Schöpfstrasse 41, 6020 Innsbruck, Austria; 2 Eurac Research, Institute for Biomedicine, Affiliated Institute of the University of Lübeck, Bolzano, Italy; 3 Department of Genetic Epidemiology, University of Regensburg, Regensburg, Germany; 4 Institute of Hygiene and Medical Microbiology, Medical University of Innsbruck, Innsbruck, Austria; 5 Department of Nephrology and Hypertension, University Hospital Erlangen, Friedrich-Alexander-Universität Erlangen-Nürnberg, Erlangen, Germany; 6 German Chronic Kidney Disease Study, Erlangen, Germany; 7 Department of Nephrology and Medical Intensive Care, Charité–Universitätsmedizin Berlin, Berlin, Germany

**Keywords:** Genome-wide association studies, Medical genetics

## Abstract

Mitochondrial DNA copy number (mtDNA-CN) is a biomarker for mitochondrial dysfunction associated with several diseases. Previous genome-wide association studies (GWAS) have been performed to unravel underlying mechanisms of mtDNA-CN regulation. However, the identified gene regions explain only a small fraction of mtDNA-CN variability. Most of this data has been estimated from microarrays based on various pipelines. In the present study we aimed to (1) identify genetic loci for qPCR-measured mtDNA-CN from three studies (16,130 participants) using GWAS, (2) identify potential systematic differences between our qPCR derived mtDNA-CN measurements compared to the published microarray intensity-based estimates, and (3) disentangle the nuclear from mitochondrial regulation of the mtDNA-CN phenotype. We identified two genome-wide significant autosomal loci associated with qPCR-measured mtDNA-CN: at *HBS1L* (rs4895440, p = 3.39 × 10^–13^) and *GSDMA* (rs56030650, p = 4.85 × 10^–08^) genes. Moreover, 113/115 of the previously published SNPs identified by microarray-based analyses were significantly equivalent with our findings. In our study, the mitochondrial genome itself contributed only marginally to mtDNA-CN regulation as we only detected a single rare mitochondrial variant associated with mtDNA-CN. Furthermore, we incorporated mitochondrial haplogroups into our analyses to explore their potential impact on mtDNA-CN. However, our findings indicate that they do not exert any significant influence on our results.

## Introduction

The intracellular energy-producing mitochondria possess their own DNA, which is a small (~ 16.6 kb in humans), circular and multi-copy genome. It encodes 37 genes for proteins of the mitochondrial respiratory complexes, mitochondrial transfer RNAs and ribosome-coding RNAs, which are essential components of its own translational apparatus^1^. We and others showed previously that alterations in mitochondrial DNA copy number (mtDNA-CN) are associated with various diseases^2–5^. Therefore mtDNA–CN has been proposed as a potential biomarker for mitochondrial dysfunction^6^. However, it is still a matter of debate whether these alterations are a cause or consequence of these diseases.

The abundance of mtDNA greatly varies between tissues, developmental stage and individuals^5,7^. The precise mechanism of its regulation is still unclear. More than 1100 mitochondrial genes are encoded in the human nuclear DNA^8^, including components of the replication and repair machinery, hence a nuclear contribution to the regulation of mtDNA-CN is strongly hypothesized^9,10^. In fact, several nuclear genes have been shown to influence mtDNA-CN^11–18^ and interestingly, mtDNA-CN has been further associated with nuclear DNA methylation patterns and nuclear gene expression^19,20^.

The growing interest in mtDNA-CN regulation has led to an increasing number of genome-wide association studies (GWAS) on mtDNA-CN^11–18^. One of the first major GWAS by Cai et al.^12^ used the mtDNA-CN extrapolated from low-coverage whole genome sequencing data (mean coverage of mtDNA 100x) in 10,442 Han Chinese females, where they identified two variants involved in mtDNA-CN regulation^12^. In another study of similar size using a quantitative PCR (qPCR)-based approach to measure mtDNA-CN, one of the identified variants was partially confirmed, but without reaching genome-wide significance^11^. With the availability of larger cohorts such as the UK Biobank (UKB), mtDNA-CN estimated from microarray intensity data rather than qPCR data are being used more often. Using microarray data, Hägg et al. identified 50 associating SNPs in UKB, 38 of which were replicated in another study^15,21^. Longchamps and colleagues performed a GWAS for microarray-based mtDNA-CN in 465,809 samples from UKB and found even more autosomal SNPs to be associated. The strongest association was found for *LONP1* (p = 3 × 10^–141^)^15^. Chong et al. showed that the correlation between mtDNA-CN estimated from microarray data and qPCR varied between r = 0.53 and r = 0.70, depending on the ancestry^17^.

We were interested in identifying loci associated with qPCR-measured mtDNA-CN, and to assess whether the findings from microarray-based approaches were equivalent with qPCR-based measurements of mtDNA-CN. In the present study, we therefore attempted to identify nuclear and mitochondrial variants modulating mtDNA-CN. We measured mtDNA-CN using a plasmid-normalized qPCR assay^22^ in more than 16,000 individuals in three highly standardized, independent studies, the GCKD, CHRIS and AugUR studies, and conducted a GWAS meta-analysis to determine variants associated with the trait.

## Methods

### Study populations

#### GCKD study

The German Chronic Kidney Disease (GCKD) study is a previously described prospective cohort study^3,23^ of 5217 adult patients with chronic kidney disease (reduced glomerular filtration rate and/or proteinuria) under regular care by nephrologists. Trained personnel obtained information on socio-demographic factors, medical and family history, medications and health-related quality of life through standardized questionnaires. Data were collected and managed using the cloud-based web platform Askimed (https://www.askimed.com). In our analysis, 4692 unrelated individuals with available mtDNA-CN and genomic data were included.

#### AugUR study

The AugUR study (Altersbezogene Untersuchungen zur Gesundheit der University of Regensburg) is a population-based cohort study of the elderly population of Regensburg (Germany) to investigate age-related traits at the genetic and non-genetic levels. Details of the study design and data collection have been described elsewhere^24,25^. Briefly, 2449 participants with at least 70 years of age at the time of sample and data collection were included. Information on sociodemographic data, lifestyle, metabolic parameters, medication intake, and morbidities was collected. The recruitment phase was split in two parts, henceforth referred to as “AugUR1” (n = 1133) and “AugUR2” (n = 1316) study. Related individuals were excluded for further analysis.

#### CHRIS study

The Cooperative Health Research in South Tyrol (CHRIS) study is a longitudinal population-based study from South Tyrol (Italy) investigating the molecular basis of health and disease in the general population. Detailed information about medical history and medication were collected by means of interviews and self-administered questionnaires. At the time of this analysis, the study comprised 9778 participants aged 18 to 94 years, with 9320 included in our GWAS (corrected for relatedness). Further study details are published elsewhere^26,27^.

#### Approval by ethics committees

Participation was based on written informed consent. All studies were carried out in accordance with approved guidelines and in compliance with current national and EU regulations, the tenets of the Declaration of Helsinki and its later amendments. The GCKD study was approved by the Ethics Committees of all participating institutions and is registered in the national registry for clinical studies (DRKS 00003971). The AugUR study was approved by the Ethics Committee of the University of Regensburg (vote 12-101-0258). The CHRIS study was approved by the Ethical Committee of the Healthcare System of the Autonomous Province of Bolzano (protocol no. 21/2011). The project “Variazioni del numero di copie del DNA mitocondriale: mutazioni e suscettibilità alle malattie” (PI: Andrew A. Hicks) was approved by the same committee (protocol no. 10/2016). The CHRIS Access Committee authorized the analysis of data and samples for this project (application no. 69).

### DNA extraction and mtDNA copy number measurement

In all three studies, biospecimens were collected following a standard protocol and samples were shipped under temperature-controlled conditions for further analyses. DNA was extracted from frozen EDTA-blood samples using the Chemagic Magnetic Separation Module I (PerkinElmer Chemagen Technologie GmbH, Germany), an automated magnetic beads-based method in GCKD and CHRIS. Within the AugUR study, part of the DNAs (> 82% of AugUR1) was extracted with reagents from Puregene (Qiagen, Hilden, Germany) and the other part (entire AugUR2) with a similar salting out method to enhance the yield in this elderly study sample. DNA was available from 4812, 2439 and 9364 participants in the GCKD, AugUR and CHRIS study, respectively.

The mtDNA-CN measurements from all three studies were performed in triplicate with the same method and in the same laboratory (Medical University of Innsbruck). No modifications to the original protocol for quantification of mtDNA-CN per diploid cell were made^28^. Briefly, we applied a duplex quantitative PCR assay that allows for simultaneous targeting of the single-copy nuclear gene beta-2-microglobulin (B2M, 86 base pairs) and the mitochondrial tRNA^Leu^ gene (108 base pairs). A region of mtDNA-tRNA^Leu^ was amplified using the forward primer 5′-CACCCAAGAACAGGGTTTGT and the reverse primer 5′-TGGCCATGGGTATGTTGTTA; a region of B2M was amplified using the forward primer 5′-TGCTGTCTCCATGTTTGATGTATCT and the reverse primer: 5′-TCTCTGCTCCCCACCTCTAAGT. Probe sequences were: FAM-5′-TTACCGGGCTCTGCCATCT-BHQ1 for tRNA^Leu^ and Yakima Yellow-5′-CAGGTTGCTCCACAGGTAGCTCTAG-BHQ1 for the nuclear gene. The qPCR was performed on a QuantStudio™ 6 Flex system instrument (Thermo Fisher Scientific, Waltham, MA, USA) using the following conditions: 95 °C for 3 min for initial polymerase activation, 40 cycles of 95 °C for 15 s and 60 °C for 1 min. The mtDNA-CN was calculated using the ΔΔCq (quantification cycle) method: 2 × E^−(ΔCq sample−ΔCq plasmid)^, where “E” is the average mean efficiency of the PCR of the PCR reaction of the two targets^29^ and “2” is the account for the two copies of nuclear DNA in a cell. In each run, a plasmid containing both targets was included to correct for inter-assay variability. In each qPCR plate, two DNA samples were included and used for monitoring the performance of the assay over the entire project.

### Genotyping data and imputation

Genotyping was performed using different platforms: Illumina Human OmniExpressExome and OMNI 2.5Exome chip array on subjects from the CHRIS study, OMNI 2.5Exome BeadChip in the GCKD study and the Illumina Global Screening Array v1/v3 in the AugUR Study. Before imputation, genotype quality control was implemented using standard parameters suggested by the calling software GeneCall by Illumina. Genotypes of all three studies were imputed based on the Haplotype Reference Consortium (HRC)^30^ on the genome build GRCh37. SNPs with low imputation quality scores (< 0.3) were excluded.

### Genome-wide association studies and meta-analyses

**Figure 1:**
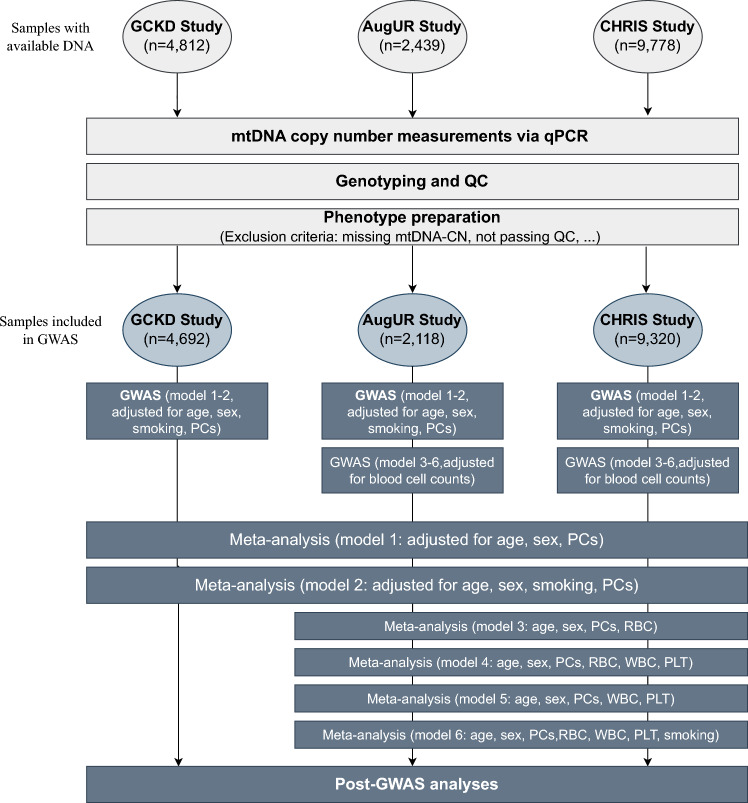
Experimental workflow of GWAS based on mtDNA-CN. DNA samples of 16,615 individuals was available for mtDNA-CN measurements via qPCR and genotyping from three studies (GCKD, *German Chronic Kidney Disease*; AugUR, *Altersbezogene Untersuchungen zur Gesundheit der University of Regensburg*; CHRIS, *Cooperative Health Research in South Tyrol*). After excluding those without determinable mtDNA-CN and those not passing the quality control (QC), GWAS was conducted for all three studies and additionally, a meta-analysis was performed in 16,130 individuals followed by post-GWAS analyses. Adjustment models are described within the flowchart using age, sex, principal components (PCs), erythrocyte counts (RBC), white blood cell counts (WBC), platelets (PLT), and smoking as covariates.

Quality control and GWAS were performed using our REGENIE^31^ based in-house Nextflow pipeline nf-gwas (version v0.3.5, available at https://genepi.github.io/nf-gwas/)^32^. The REGENIE algorithm performs two steps: (1) fitting a whole genome regression model to account for population structure and relatedness using all included genotyped variants pruned for linkage disequilibrium (LD; 1000 variant window, 100 step size, r^2^ < 0.9)) and (2) single-variant association testing conditioned on predictions made in step 1^31^. Inverse normal transformed mtDNA-CN was used as the outcome variable of the regression model with different sets of covariates. In addition, ß-estimates for top hits are given on the original scale of mtDNA-CN for easier interpretation of results. Covariates were selected based on correlation structure with the outcome variable (mtDNA-CN) and among themselves. We tested the correlation of age, sex, smoking (current smokers vs. former- or never-smokers) and blood counts (erythrocytes, leukocytes, platelets) in each study via Pearson correlation and corresponding correlation plots can be found in Fig. S1. GWAS analysis was performed on HRC-imputed data with additive genetic effect in six different adjustment models for each study, stratified by sex and stratified by smoking status (never smoker, former smoker and current smoker). Base covariates (model 1) included in the study were age, sex and the first four genetic principal components (PC), while model 2 included all covariates from model 1 plus smoking status. GWAS analysis with models 1 and 2 were performed in each study. Model 3–6 (see Fig. 1 for details) included additional adjustments for blood cell counts that were not available in the GCKD study—therefore, model 3–6 were only performed in the CHRIS and AugUR studies. In CHRIS, we corrected for potential batch effects (three different genotyping batches). In AugUR, we analyzed AugUR1 and AugUR2 separately as two independent studies since the different DNA extraction methods used in AugUR1 and AugUR2 could have influenced mtDNA-CN measurements^28^. For each individual study and model, we determined the genomic control inflation factor lambda (range: 0.939–1.001) visualized in QQ-plots. Since inflation was hardly present, no GC correction was applied. The meta-analysis based on inverse-variance weighted GWAS summary statistics was performed using metal^33^. Heterogeneity was determined using I^2^ measurements. A schematic summary of the simplified workflow is depicted in Fig. 1.

Statistical significance level was set at p < 5 × 10^−8^. Phenotype data preparation, post-GWAS analysis and other additional analyses were performed using R, version 4.2.1 (R Foundation for Statistical Computing, Vienna, Austria).

### Post-GWAS analyses

Post-GWAS analyses included QQ-plots (including genomic-control inflation factor lambda values, range of meta results 0.960–0.988) and Manhattan plots (custom scripts using R), including variants with minor allele frequencies (MAF) > 0.001. Locus regions were defined as ± 250 kb around the lead SNP and regional plots of the loci were generated using the LocalZoom platform^34^ (available at: https://statgen.github.io/localzoom/). To identify independent signals per locus, stepwise conditional analyses were performed with GCTA-COJO in regions ± 250 kb around the lead SNP^35^ and for this, we used CHRIS genotypes as the reference for LD. The variance explained by the significantly associated loci (± 250 kb around the lead SNP) was determined using GCTA-GREML (version 1.93.2)^35^. The UCSC Genome Browser^36^ and its implementations were used for functional annotation of associated variants. For sex-stratified analyses, we tested differences in effect estimates between male and female individuals using a z-test of difference^37^ (see Supplement for more details). The same approach was applied for smoking-stratified analyses between current smokers, former smokers and never-smokers (p-value cut-off = 2.5 × 10^–8^ due to comparison of three groups).

To determine if our top hits are already linked to other traits, we conducted a SNP lookup using PhenoScanner^38,39^. To further explore the impact of blood cell counts (erythrocyte counts (RBC), white blood cell counts (WBC), platelets (PLT)) and smoking on mtDNA-CN, we performed a mediation analysis using the mma package^40^ for our top hits. Before conducting the mediation analysis, we first assessed whether the necessary conditions for potential mediators were met. Only potential mediators that showed a significant association between the SNP and mediator, as well as between the mediator and outcome, were included in the analysis. Variables that did not meet these conditions were included in the model as covariates. Mediation models were further adjusted for age, sex and 4 PCs. Mediation analysis was carried out in studies with available blood cell counts separately (CHRIS and AugUR). The proportion mediated was summarized using a sample-size weighted average.

Colocalization analyses were conducted to investigate whether the identified variants associated with mtDNA-CN also influence gene expression levels. Meta-GWAS p-values were plotted against whole blood eQTL p-values of the same SNPs, taken from the eQTLGen Consortium^41^ (n = 31,684 from cis-eQTL data). Genes within a window ± 250 kB around the GWAS top hit were included, and those showing a false discovery rate (FDR) < 0.05 in the association with expression are reported. For each region, posterior probability of the five hypotheses (H0–H4) was evaluated. Signals with high H4 posterior probability (> 70%) were deemed to have strong evidence of colocalization with the same potentially causal variant. Based on the genes identified from the colocalization analysis, we investigated protein–protein interactions using the String database^42^.

### Mitochondrial haplogroup determination and mitochondrial variants

In order to determine the mitochondrial haplogroups, the mitochondrial genotypes were first quality-controlled including allele checks against the revised Cambridge Reference Sequence (rCRS; NC_012920.1, 16,569 bp). The genotypes from five different microarrays were assessed for their performance of haplogroup classification. Each microarray was simulated based on full mtDNA sequences corresponding to all haplotypes present in the global human phylogenetic mtDNA tree Phylotree 17^43^ and masked to keep only the genotypes of the corresponding microarrays to calculate its performance for accurate haplogroup assignment. Subsequently the mitochondrial haplotypes from 16,130 samples were converted from the PLINK to VCF format with PLINK 2.0^44^ with subsequent quality filtering for missing genotypes in VCFtools^45^ (call rate ≥ 95%). We estimated the haplogroups using HaploGrep 2 (version 2.4)^46^ with the “–chip” option in 16,121 samples. The five microarrays cover varying mtDNA variants ranging from 140 on the GSAMD v1 Chip to ~ 1400 on the GSAMD v3. Since therefore different haplogroup resolutions are expected, we grouped the samples in five consensus groups: (1) R0 including haplogroups R0, H, V and HV (51.6%), (2) JT including macrohaplogroups J and T with all sub-haplogroups (20.2%), (3) UK including all U and sub-haplogroups including K (21.5%), other Europeans with haplogroup N1, N2, X (6.0%) and non-Europeans containing the remaining 0.7% of haplogroups (A, B, D, G, L, M, N8, R9). Given the vast difference in mtDNA variants covered on the five different microarrays, we used MitoImpute^47^ with the Reference Panel v1 0.01 (MAF 1%) to infer missing mtDNA variants. In short, MitoImpute runs the chromosome X imputation pipeline via IMPUTE2 with no recombination (thereby artificially considering all samples as males) on a globally diverse Reference Panel (n = 36,960). We assessed the results based on haplogroups estimated prior and after imputation with HaploGrep 2. Here the phylogenetic distances between the haplogroups calculated with the “distance” parameter were analyzed. This imputation step increased the shared variants on the five different microarrays from 27 to 413 variants (Fig. S2). The VCF files were normalized by splitting multi-allelic sites into separate rows and reference allele mismatches were fixed with BCFtools^48^ *norm*. Allele frequencies were compared with Helix's mitochondrial variant database (available at helix.com/mito) as reference (Pearson > 0.9).

Besides using the mitochondrial haplogroups as a covariate in the GWAS, we also tested whether the mtDNA-CN differed between the haplogroups via a linear mixed-effects model accounting for the different studies and correcting for age and sex.

GWAS on mitochondrial variants was performed in R using the vcfR package (version 1.13.0)^49^ and linear regression models for each variant were calculated. Results were meta-analyzed using the metafor package^50^ (using random-effects models, fitted by REML estimation). Heterogeneity was tested using I2 measurements. Results of 305 mitochondrial variants, which were present in at least two studies, were included. P-values below 0.00016 (0.05/305) were considered significant.

Genome-wide significant nuclear variants and those close to genome-wide significance (cutoff p < 1 × 10^–7^) were annotated with MitoCarta3.0^8^. For all models, the results from the meta-analyses were augmented with the number of all entries and unique gene names in MitoCarta3 as well as the pathways via an R-script. Additional mitochondrial sub-compartments were analyzed for all genes with the COMPARTMENTS resource^51^ (see Fig. S3).

### Comparison with previous findings

Table S1 provides a summary of the main characteristics and findings from various GWAS conducted on mtDNA-CN. We selected SNPs, which were identified in the largest study based on array data from Longchamps et al.^15^. SNPs were selected using the following criteria: (1) at least two SNPs within a 1 Mb window showed genome-wide significance, (2) the lead SNP was directly genotyped and/or (3) the respective locus was significant in at least one of their three complementary analyses. We compared the concordance of these results with our results based on qPCR measurements by performing an equivalence test^52^ applying a difference margin of 0.5, corresponding to 0.5*variance of the phenotype (see Supplement for more details).

## Results

### Sample characteristics

In total, 16,130 individuals of European ancestry were included in the meta-analyses while correcting for relatedness. Baseline characteristics of participants from all three studies are presented in Table 1. Half of all participants (50.3%) were female. The age-range of all participants was between 18 and 95 years (mean age: 54.3 years). Inter-assay mtDNA-CN coefficients of variation of the two control samples included in each qPCR plate to monitor performance in the 191 independent experiments were 6.0% and 8.3%.

**Table 1:** Baseline characteristics and genotyping information of all individuals included in the meta-analysis.

	GCKD	AugUR	CHRIS
N^a^	4692	2118	9320
Sex (female)	1862 (39.7%)	1112 (52.5%)	5143 (55.2%)
Age^b^	60.2 ± 11.9	78.3 ± 5.0	45.8 ± 16.3
Current Smoker	746 (15.9%)	112 (5.3%)	1632 (17.5%)
Leukocyte count (10^3^/µl)^b^	NA	6.5 ± 2.0	6.2 ± 1.8
Erythrocyte count (10^6^/µl)^b^	NA	4.5 ± 0.4	4.9 ± 0.5
Platelet count (10^3^/µl)^b^	NA	240 ± 62	254 ± 57
Mean mtDNA-CN^b^	107.3 ± 36.5	150.9 ± 43.9	143.5 ± 51.1
DNA source	Whole blood	Whole blood	Whole blood
DNA extraction	Automated magnetic beads-based method	Manual salting out method	Automated magnetic beads-based method
mtDNA-CN measurement	qPCR	qPCR	qPCR
Genotyping array	Illumina Infinium^®^ OMNI 2.5Exome	Illumina Infinium^®^ Global Screening Array (v1/v3)	Illumina Infinium^®^ Human OmniExpressExome, Omni 2.5Exome
Genotype quality control (exclusion criteria)	HWE p < 1 × 10^–5^; sample call rate < 0.97, SNP call rate prior to imputation < 0.96	HWE p < 1 × 10^–8^; call rate < 0.95; monomorphic variants	HWE p < 1 × 10^–6^; call rate < 0.98; monomorphic variants
Imputation	HRC	HRC	HRC

^a^n refers to individuals included in the genome-wide association studies.

^b^Mean ± standard deviation.

### Genome-wide association study: two loci associated with mtDNA-CN

**Figure 2:**
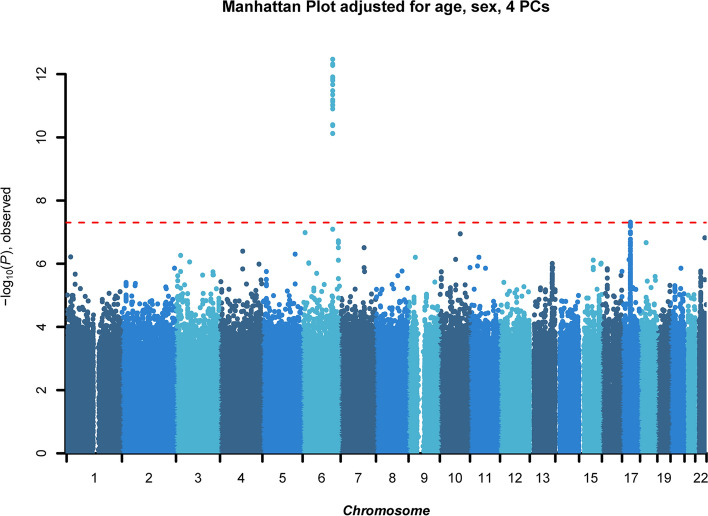
Manhattan plot illustrating genome-wide autosomal associations for mtDNA-CN in all three studies (GCKD, AugUR, CHRIS), adjusted for age, sex, and four principal components (PCs). The red line represents the threshold for genome-wide significance (p-value < 5 × 10^−8^). The x-axis gives the chromosomes, the y-axis shows the –log10 p-values of imputed SNPs.

We found genome-wide significant variants for the main model (Fig. 2) and further adjusted models (Fig. S4). The meta-analysis revealed three significant loci associated with mtDNA-CN based on the main model adjusted for age, sex and the 4 PCs. We identified a genome-wide significant locus located on chromosome 6 (lead SNP: rs4895440, ß = 0.09, 95%CI = 0.06–0.11, p = 3.39 × 10^–13^). The effect on the original scale equals an increase of 3.31 mtDNA copies per effect allele. The lead SNP is intergenic between the *HBS1L* and *MYB* genes with several SNPs in LD (Fig. S5a). This variant remained genome-wide significant and showed similar ß-estimates when additionally adjusting for smoking and erythrocyte count, but the effect decreased after adjusting for white blood cell and platelet count (see Table S2 for more details). A second genome-wide significant locus on chromosome 17 is represented by a frequent missense variant within the *GSDMA* gene (rs56030650, ß = − 0.06, 95%CI = − 0.08; − 0.04, p = 4.85 × 10^–08^; Fig. S5b). On the original quantification scale, this variant was associated with − 2.55 mtDNA copies per effect allele. This association was genome-wide significant in the age-, sex- and 4 PC-adjusted model, but we noticed a reduction of the ß-estimate only after adjusting for leukocytes and platelets (Table S2). However, the estimate remained stable when adjusted for smoking and/or erythrocyte counts. The explained genetic variance by these two loci (± 250 kb around the lead SNP) was 2.45% (standard error 0.7%) in the main model. In both loci, no heterogeneity between studies was observed (I2 = 0).

A third locus (rs9306373) was identified around *TSPO* with stable ß-estimates over different adjustment models (ß = − 0.15, 95%CI = − 0.20; − 0.10, p = 9.89 × 10^–09^ when adjusted for age, sex, PCs, platelets and leukocyte counts). However, the effect was mainly driven by the CHRIS study (Table S3). We observed highly different MAF in CHRIS (0.07 compared to 0.004–0.005 in the other studies), observed a ß-estimate in the opposite direction solely in CHRIS and all together, this variant exhibits a high level of heterogeneity (I2 ≥ 50%, p = 0.003). To identify the source of possible heterogeneity, we checked the cluster plots for the specific *TSPO* variant, provided by the Illumina GenomeStudio software (Fig. S6), and we found highly noisy cluster distribution of the A/B normalized intensity, leading to an increase in the genotype call uncertainty for two out of three of the CHRIS genotyping batches. We concluded that this finding might thus be a technical artifact, and excluded the locus from further consideration.

Stepwise conditional analyses did not reveal any further independent signals at each of the identified loci. The results confirmed the two lead variants described above, with no hidden conditioning signal. Details on all three genome-wide significant variants are provided in Table 2.

**Table 2:** List of genome-wide significant autosomal top hits from the meta-analysis.

Chr	Position	Lead SNP	A1	A2	Freq A1^a^	Freq A1 (1000G)^b^	Effect	StdErr	Nearest gene	Distance BP	N	Best P-value	Proportion of variance explained by this SNP	Adjustment
6	135426558	rs4895440	t	a	0.2686	0.2773	0.0855	0.0118	*HBS1L*	50,522	16,130	3.39 × 10^–13^	0.0032	Age, sex, 4 PCs
17	38131187	rs56030650	a	c	0.4305	0.4573	− 0.0573	0.0105	*GSDMA*	0	16,130	4.85 × 10^–08^	0.0018	Age, sex, 4 PCs

^a^Freq A1 = weighted average of frequency for allele 1 across all studies based on our meta-analysis results.

^b^Freq A1 (1000G) = frequency of the A1 allele based on 1000 Genome Europeans.

### Association with gene expression and colocalization analysis

In the association with expression levels, eight genes were found to have a FDR of < 0.05 with the respective GWAS lead SNP, two of them in the chromosome 6 gene region, six on chromosome 17. Colocalization analysis thus revealed either high H3 probability, that is, association with both expression and mtDNA, but not the same variant that is potentially causal, or H4, with indication for even the same potentially causal variant (summarized in Table S4). For *MYB*, in the chromosome 6 region, there is strong evidence for colocalization with the same potentially causal variant (H4 = 0.98). In contrast, *HBS1L* shows a H3 probability close to 1, indicating that the GWAS and eQTL signals are driven by different genetic variants. In the second region on chromosome 17, potential colocalization between the GWAS signals and eQTL signals was observed for *GSDMB* and *ORMDL3.* Interestingly, the GWAS top hit corresponds to the lead SNP in the eQTL data for both of these genes. For other genes, including *MED24*, results are less conclusive. The H3 probability for *MED24* is 0.8, while the H4 probability is 0.2. However, visually comparing both peaks shows that they are in the same region and that the lead SNPs of the eQTL and GWAS are in LD. Results of colocalization analyses for all included genes are visualized in Figs. S7 and S8.

Based on the results of the colocalization analysis, we utilized the String database^42^ to explore protein–protein interactions. As shown in Fig. S9, this investigation indicated the involvement of MED24, PSMD3, GSDMA and HBS1L in measured mtDNA-CN. Additionally, several of the genes were found to be associated in some type of blood cell composition.

### Analysis stratified for sex and smoking status

When we performed each GWAS model stratified for male and female individuals, we identified a genome-wide significant locus which was present in only one of both sexes (men n = 8012, women n = 8118). While we could not identify genome-wide significant variants in the sex-stratified analysis in the main model, we found a significant locus in our cell count-adjusted analysis (without GCKD). In females, a significant association between a rare variant in *DIPK1B* and mtDNA-CN was found (lead SNP: rs186793011, p = 4.21 × 10^–08^; ß = 0.70 in women and − 0.15 in men; MAF 0.005/0.006). Details are provided in Table S5. A z-test comparing effect estimates between male and female individuals found no significant difference between both sexes for any of the variants in any of the adjustment models (on genome-wide significance level), though.

When stratified for smoking status (adjusted for age, sex and 4 PCs), we identified no significant difference between current smokers, past smokers and non-smokers. In all three groups, ß-estimates for top hits had the same effect direction (Table S6). Further, no additional variants were identified in any of the subgroups.

### Mitochondrial DNA variants

**Figure 3:**
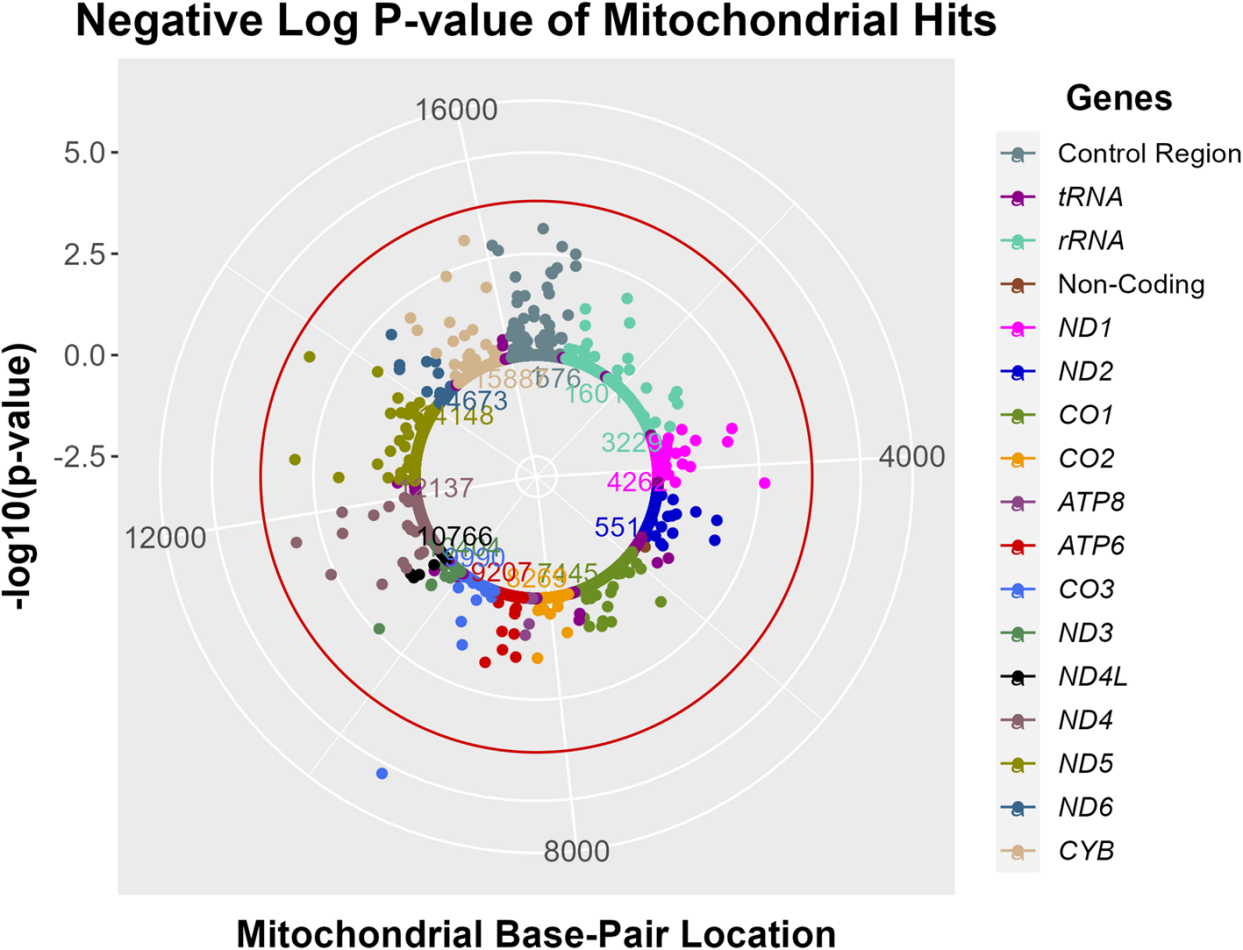
Results of meta-analysis on mitochondrial variant associated with mtDNA-CN. In this solar plot, mitochondrial variants are colored based on the genomic region (see legend). Mitochondrial base pair location is shown as numbers in the inner circle, association p-values (-log10 p-value) are illustrated on the y-axis (representing the distance between the inner circle and the outer circles). The threshold for significance (red circle) was set at < 0.00016 based on the number of variants included in the analysis (0.05/305).

Meta-analysis of imputed mitochondrial variants revealed one rare genome-wide significant variant (MT:9548_A, MAF = 0.0003, ß = − 1.30, p = 5.61 × 10^–06^) when adjusted for age, sex and 4 PCs (p = 0.00013 when additionally adjusted for smoking status). Several common variants were close to reaching the significance threshold of 0.00016 (e.g. MT:13708_A, n = 1880, p = 0.00047) as visualized in a solar plot (Fig. 3). All mitochondrial variants with p-values below 0.001 are listed in Table 3.

**Table 3:** List of mitochondrial variants with p < 0.001 in the main model of the meta-analysis.

Position	Freq	Freq (HelixMTdb)	Effect (main model)	StdErr (main model)	I^2^	N	P-value (main model)^a,b^	P-value (adjusted as main model + smoking)^b^
MT:9548_A	0.002	0.009	− 1.298	0.286	0	12	5.61 × 10^–06^	1.00 × 10^–04^
MT:13708_A	0.101	0.102	0.091	0.026	0	1880	4.72 × 10^–04^	1.12 × 10^–02^
MT:11719_A	0.500	0.590	0.053	0.016	0	8899	7.32 × 10^–04^	9.00 × 10^–04^
MT:73_G	0.520	0.612	0.053	0.016	0	8994	7.57 × 10^–04^	7.00 × 10^–04^
MT:15784_C	0.007	0.016	− 0.317	0.095	0	112	8.12 × 10^–04^	1.16 × 10^–02^
MT:12612_G	0.095	0.081	0.087	0.027	0	1779	1.08 × 10^–03^	1.23 × 10^–02^

^a^Main model: adjusted for age, sex, 4 PCs.

^b^P-values below 0.00016 (0.05/305) were considered significant. Bold font indicates significance.

### Association between mitochondrial haplogroups and mtDNA copy number

Mitochondrial haplogroups in each study were determined using HaploGrep 2^46^. As expected, the most common mitochondrial haplogroups belonged to typical European mitochondrial lineages (see Fig. S10 for haplogroup distribution in each study). Considering the different studies, linear mixed-effects models revealed significant differences for mtDNA-CN (on original scale) between R0 and JT (ß = 3.05, p = 0.0012) and R0 and UK (ß = 1.91, p = 0.039), however, no difference to the other haplogroup clusters was seen. In a sex-stratified analysis, we found significant differences between R0 and JT (ß = 5.65, p < 0.001) and R0 and UK (ß = 3.94, p = 0.0016) in males. In females, we did not identify a significant difference for mtDNA-CN between any of the haplogroups (see Fig. S11 for distribution of mtDNA-CN between haplogroups stratified by sex).

Besides comparing mtDNA-CN between haplogroups, we also added haplogroups as covariate in our GWAS by setting the most common clade R0 as the reference. This did not change the results of the meta-analysis.

### Adjustment for blood cell counts and mediation analyses

It is well investigated that mtDNA-CN measurements in peripheral blood are influenced by the blood cell counts^17,53^. We performed additional sensitivity analyses by bringing leukocyte and thrombocyte count into the equation as described by Hurtado-Roca and colleagues^53^. This resulted in similar results without major changes and both variables (original mtDNA-CN vs. mtDNA-CN adapted by Hurtado equation) showed high correlation (r = 0.96). Additionally, we applied various adjustment models including adjustment for blood cell count parameters (model 3–6). Information on blood cell counts was only available for AugUR and CHRIS, but not for the GCKD study, which reduces the sample size to 11,438. In Table S2, we additionally present our main model without the GCKD study in order to illustrate the impact of reduced sample size on the estimates. Our findings indicate that the exclusion of GCKD, with the subsequent reduction in power, has only a minor influence on the effect estimates. When using blood cell counts as covariates in the GWAS, we observed lower effect sizes compared to models not adjusted for blood cell composition (exception: sex-stratified analyses).

**Figure 4:**
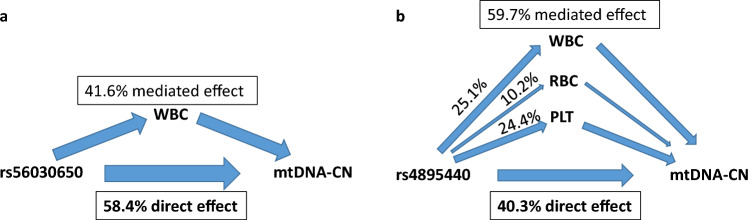
Visualized results of the mediation analysis examining the influence of the two GWAS top hits on mtDNA-CN through mediator variables: erythrocyte counts (RBC), white blood cell counts (WBC), platelets (PLT). Two studies, AugUR and CHRIS study, were included and the shown percentages are based on weighted means by sample size. The arrows in the plot represent the direction and magnitude of the effects.

The mediation analysis revealed that the effect of rs56030650 on mtDNA-CN is mediated by white blood cell count with a proportion of 41.6% (6.6% in AugUR and 49.6% in CHRIS), meaning that still 58.4% of the SNP-effect affects mtDNA-CN directly (Fig. 4, panel a). None of the other variables met the criteria for a potential mediator for this variant.

Similarly, the mediation analysis for rs48955440 demonstrated that 40.3% of the SNP effect directly affects mtDNA-CN. The mediated effect accounted for 59.7% of the total effect and was distributed among WBC, RBC and PLT with varying proportions in AugUR and the CHRIS study. Specifically, leukocyte counts, and platelet counts made significant contributions to the mediation model, each explaining approximately 25% of the total effect (Fig. 4, panel b). Although the estimates for mediated effects and proportions differed between both studies (Table S7), the mediation models were consistent in the identified mediators for both SNPs and both studies.

### Analyses based on mitochondrial annotation

Nuclear variants identified in the meta-analysis were annotated with MitoCarta3.0^8^ to identify nuclear genes with mitochondrial localization and mitochondrial pathway contribution. We investigated whether prioritized genes associated with mtDNA-CN were enriched for mitochondrial genes/localizations and pathways. Thereby six different models where annotated with an average of 28.8 entries, in 24 unique genes identifying TSPO and FHIT (only in the main model) in MitoCarta3 Pathways (Lipid metabolism > Cholesterol, bile acid, steroid synthesis and Metabolism > Nucleotide metabolism > Nucleotide synthesis and processing respectively) shown in Tables S8–S11.

### Comparison between published results based on microarray intensity data and our current results based on qPCR data

**Figure 5:**
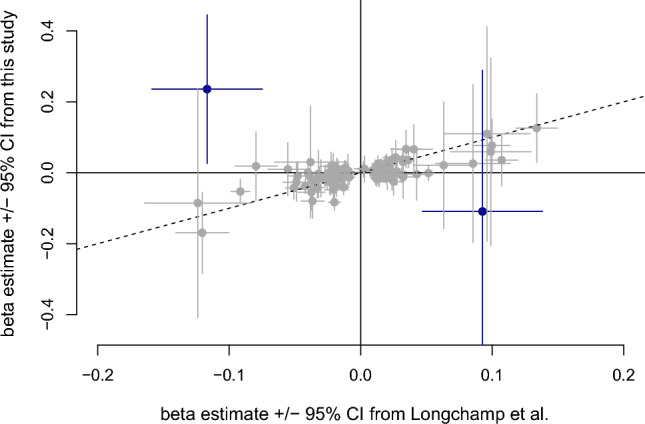
Comparison of ß-estimates ± 95% confidence interval (CI) from our study and the study by Longchamps and colleagues^15^. The ß-estimates for the two SNPs highlighted in dark blue are not equivalent using an equivalence test. Dashed line indicates line of agreement with slope 1, dotted line a linear regression line between the two estimate vectors.

Finally, we aimed to compare the consistency with previous results^15^ (n = 465,809, adjusted for age, sex and blood cell counts) estimated based on microarray intensity data with our main results measured with qPCR (covariates: age, sex, four PCs). Fourteen of the lead variants reported by Longchamps et al.^15^ were not investigated in our study due to low imputation quality, or exclusion of multi-allelic variants during quality control, and therefore, 115 variants were compared. Due to large differences in samples size, and therefore power, we aimed to evaluate the concordance of effect estimates rather than looking at genome-wide significance or replication. In 73 of the 115 SNPs, we observed consistent ß-estimate directions. A test on equivalence showed significant equivalence of ß-estimates for all but two SNPs (rs200957609 (*AP5Z1*); rs141227171 (*LIPC*), Table S11). Altogether, correlation between effect estimates is quite low (r^2^ = 0.14, p = 3.62 x 10^-05^) with systematically lower effect estimates in our study compared to Longchamps et al.^15^ (Fig. 5), which is expected, though, since SNPs were selected from the Longchamps study results (“winners curse”^54^).

## Discussion

### Main findings

In this meta-analysis using qPCR-based data from 16,130 individuals of European ancestry, we identified two nuclear gene regions, near *HBS1L/MYB* and in the *GSDMA* gene, associated with mtDNA-CN in all participants, and two loci in sex-specific analyses. We further found one rare mitochondrial variant associated with mtDNA-CN. The regulation of mtDNA-CN by genetic variants in the mitochondrial genome probably does not play a major role, since the only mitochondrial variant associated with mtDNA-CN was very rare.

To the best of our knowledge, this is the largest GWAS meta-analysis on mtDNA-CN measured by a highly standardized qPCR performed in the same laboratory for all three studies. The first genome-wide significant identified locus (rs4895440, an intergenic variant located between *HBS1L* and *MYB*) was reported to be associated with erythrocyte, platelet, and monocyte counts as well as erythrocyte volume and hemoglobin content and with sickle cell disease^55–59^. This variant was no longer significant after adjusting for white blood cell counts and platelets, which indicated an indirect association with mtDNA-CN primarily based on blood cell composition.

The second genome-wide significant locus was within the *GSDMA* gene (rs56030650, p = 4.85 × 10^–08^). This locus was significantly associated with mtDNA-CN when adjusted for age, sex and 4 PCs but was no longer significant when we adjusted for smoking and blood cell counts. However, the ß-estimate remained stable when adjusted for smoking and the effect decreased only when adjusting for platelets and leukocytes. This variant has been associated with phosphatidylcholine levels^60^. The gene is mainly expressed in human skin and is known to regulate mitochondrial homeostasis, including mitochondrial oxidative stress^61^.

The results from the co-localization analysis revealed additional gene regions on chromosome 17, namely *GSDMB*, *ORMDL3*, *PSMD3*, *MED24* and *IKZF3*. *GSDMB* (Gasdermin B) is part of the gasdermin family, as is *GSDMA*, both playing an important role in pyroptotic activity and both were reported in childhood asthma^62–64^. Analyzing functionally similar genes obtained by Genemania^65^ indicates a central role for the gasdermin protein domain (see Fig. S12) showing genetic interactions with HBS1L, MYB and co-expression of ORMDL3. In general, proteins of the gasdermin family are closely connected and they possess a two-domain structure, comprising a well-preserved N-terminal domain responsible for pore formation and a C-terminal inhibitory domain^63^. Recent work indicates that mtDNA is released through pores on the outer mitochondrial membrane formed by GSDMD-NT binding to cardiolipin. Subsequently the cytosolic mtDNA can trigger inflammation, which can result in pyroptosis^66^. A recent investigation could show the increase of cytosolic mtDNA-CN in odontoblasts suggesting that mtDNA-GSDMD-STING signaling is involved in the regulation process^67^. Miao et al^68^. found correlations between GSDMD activation in neutrophils and mtDNA plasma content in systemic lupus erythematosus patients. Similarly, a recent work also found a role of GSDMA in cell death, by targeting mitochondrial membranes, resulting in higher ROS generation, mitochondrial dysfunction as well as mtDNA release into the cytosol^69^.

*ORMDL3* has also been associated with asthma and was shown to regulate mitochondrial calcium influx^70,71^. It is thought to be involved in endoplasmic reticulum stress, oxidative stress and mitochondrial dysfunction^72^. *PSMD3* (Proteasome 26S Subunit, Non-ATPase 3) is involved in protein degradation and has previously been found to regulate mtDNA-CN in the cluster together with *MED24* (Mediator Complex Subunit 24), both of which are associated with neutrophil count^11^.

Lastly, analysis of protein–protein interactions with the String database indicated the involvement of MED24, PSMD3, GSDMA and HBS1L with the measured mtDNA-CN.

### Integration of results in the context of available literature

Several GWAS on mtDNA-CN have been published and the main characteristics and findings are summarized in Table S1. Due to the heavy workload of qPCR-based measurements of mtDNA-CN, the largest studies are based on array-based estimates. Smaller studies have measured mtDNA-CN mainly by qPCR which is still the most widely used approach.

The first GWAS was conducted by Lopez and colleagues in 386 Spanish subjects and mtDNA-CN was measured in buffy coat by qPCR^13^. The authors did not find any genome-wide significant SNP. Cai and colleagues^12^ extracted DNA from saliva of 10,442 Chinese women and retrieved mtDNA-CN from low-coverage whole genome sequencing data. The GWAS identified two loci influencing mtDNA-CN: one in the *TFAM* gene (rs11006126, p = 8.73 × 10^–28^) and one in intron 1 of the *CDK6* gene (rs445, p = 6.03 × 10^16^). The two variants were not genome-wide significant in our study, but nominally significant into the same direction (p = 0.018 and p = 0.003). Notably, mtDNA-CN is tissue-specific, and DNA was derived from saliva in Cai et al.^12^ and from blood in our study. Our top hits were not present in their list of SNPs with p < 10^–06^. Guyatt et al. conducted a GWAS in two population-based cohorts with a total of 11,253 individuals based on qPCR-measured mtDNA-CN^11^. In meta-analyses performed in different groups, no SNP reached genome-wide significance. However, two loci (p < 10^−06^) were identified from the main meta-analysis of all adult females (n = 6799) and these loci were associated with white blood cell counts^11^. Especially in these smaller studies, many different covariates were used (e.g. age, sex, smoking behavior and oral contraceptives in Lopez et al.^13^ or age and sequencing batch in Cai et al.^12^) and therefore, results are hardly comparable.

Besides these smaller, mostly qPCR-based studies, several GWAS on mtDNA-CN estimations via microarray intensity data using UKB data and additional studies have been published^15–17, 21^. For instance, Hägg et al. found 50 significantly-associating SNPs in UKB (n = 295,150), and 38 of those were validated by Longchamps and colleagues as genome-wide significant in an even larger sample set including the UKB and the CHARGE consortium (n = 465,809)^15,21^. Longchamps and colleagues found 129 SNPs to be associated with mtDNA-CN, the strongest one being *LONP1* (rs11085147, p = 3.00 × 10^–141^). Interestingly, neither this exact variant (p = 0.003) nor another variant (p ≥ 0.003) in this locus was significant in our studies. In their GWAS, they also identified *TFAM* (rs12247015, p-value = 1 × 10^–55^), which, however, also did not reach genome-wide significance in our study (p-value = 0.0004). However, we also did not find major differences, as equivalence tests between our results and Longchamps et al.^15^ only highlighted two SNPs as not being equivalent. In a recent study utilizing whole-genome sequencing data (n = 30,666)^73^, no variants of genome-wide significance were identified. However, Zaidi et al. also conducted a comparison with the findings of Longchamps et al., where only three variants showed significant replication. Similar to our own equivalence test, the effect sizes observed in their study correlated to those reported by Longchamps et al. Another large GWAS identified new loci and revealed the involvement of *SAMHD1* mutation status on mtDNA copy numbers as well as an association with genes of mtDNA depletion disorders^17^. Gupta et al. performed a GWAS using blood cell-adjusted and non-corrected mtDNA-CN (n = 274,832)^18^. They identified 92 nuclear loci associated with mtDNA-CN. While our two main loci showed high significance in their unadjusted mtDNA-CN GWAS, the significance of these signals weakened after covariate adjustment including blood cell counts. Furthermore, Hägg et al.^21^ provided a list of genes found to be significantly accumulated with mtDNA abundance associated variants (provided in their Supplementary Table S6). Within each of the top four genes of that list (*MED24*, *CSF3*, *PSMD3* and *GSDMA*), we also found at least one SNP with p-values < 3 × 10^–7^. Therefore, differences compared to previous studies might most likely be explained by less power. However, generally smaller effect sizes in qPCR measurements compared to array-based estimations have also been shown^74^.

So far, only a few publications are available on mitochondrial variants that regulate the variation of mtDNA levels^12,15^. Cai et al. investigated mitochondrial variants and identified position 513 (p = 3.27 × 10^−9^) as significantly associated with mtDNA-CN which was not significant in our study (p = 0.09)^12^. Longchamps et al. used mitochondrial variants to investigate the relations between mitochondrial function and mtDNA-CN associated traits^15^.

### Relevance of covariate adjustment

As age and sex definitely influence mtDNA-CN in blood, we adjusted for these two parameters in all models. Moreover, we chose smoking as an adjustment variable which often is shown to influence mtDNA-CN^75^, however, in our study it had only minor effects on ß-estimates. In contrast, the role of blood cell counts on mtDNA-CN is not completely clear, but has been shown several times in the past as one of the most important factors affecting mtDNA-CN. We therefore chose additional models adjusting for blood cell composition. While adjusting for different blood cells had an effect on our outcomes, applying the suggested formula by Hurtado et al. did not influence our results.

Several of the studies listed in Table S1 did not incorporate the blood cell counts in their final analyses, which makes comparison of results difficult. On one hand, GWAS studies are usually adjusted only for age, sex and principal components, since confounding is usually not an issue for typical GWAS. Further adjustments are often performed only as sensitivity analyses. On the other hand, for mtDNA we might have a special situation which is sometimes discussed controversially since blood cell counts are covariates of interest, as the composition of blood cells can potentially cause misinterpretation of results if not accounted for. Particularly in studies examining associations between mtDNA-CN and specific phenotypes, it is advisable to adjust especially for leukocytes as they may act as a potential mediator. Different blood cell types possess varying levels of mtDNA. For instance, if a specific blood sample contains a higher proportion of leukocytes with elevated mtDNA-CN, it could artificially inflate the overall measurement of mtDNA copy number. The same applies to high levels of thrombocytes, as these cells lack a nuclear genome. However, by adjusting for blood cell counts we might miss genes which have an influence on blood cells and secondarily on mtDNA. Performing a two-step approach using first the typical GWAS adjustments followed by an adjustment for blood cell count might contribute to a better understanding how the final measurement of mtDNA-CN is influenced. Regardless, it is still a controversy whether blood cell composition has to be considered as covariate in GWAS on mtDNA-CN since GWAS are very unlikely to be prone to confounding.

While there may be a debate regarding the necessity of adjusting for blood cell counts in GWAS, we wanted to assess the extent of which the effect truly originated from mtDNA-CN. Thus, in the present study, we investigated the mediation effects through leukocytes, erythrocyte counts, and platelets on the relationship between our two top hits and mtDNA-CN. Our findings revealed that even though a substantial proportion of the total effect was mediated through blood cell composition, rs56030650 and rs4895440 exerted a significant direct effect of 58.4% and 40.3% on mtDNA-CN, respectively. The observed direct effect suggests that both variants have a direct impact on mtDNA-CN independent of its influence through mediators. Additionally, the identification of these mediators provides valuable insights into the underlying mechanisms through which these variants influence mtDNA-CN and once again, highlights the complex nature between mtDNA-CN, its genetic regulation and blood cell composition.

These findings from the mediation analysis were in line with a SNP lookup in a GWAS on various blood cell traits including > 170,000 individuals^76^, confirming that both of our lead SNPs have previously been associated with blood cell count traits. In this study, rs4895440 on chromosome 6 was primarily associated with RBC, platelet counts and WBC. rs56030650 showed associations primarily with WBC, while neither red blood cell counts, nor platelet counts were associated with this variant.

### Strengths and limitations

Over the last few decades, qPCR has been the gold standard for quantification of mtDNA-CN. However, in large studies including several hundreds of thousands of participants, this approach is no longer considered feasible. Therefore, alternatives based on whole exome/genome sequencing and microarray-based methods for mtDNA-CN estimation were developed^74^. The correlation between array-based mtDNA-CN estimates and qPCR-derived copy numbers varies between pipelines used for data analysis: e.g. the MitoPipeline^77^ shows a correlation coefficient of ~ 0.5 whereas AutoMitoC^17^ was validated in almost 5800 samples using our qPCR approach^28^ and found a higher correlation between both methods (r = 0.64; p < 2.23 × 10^–308^). Although there are several practical reasons to choose array-based mtDNA-CN estimates, we argue that our qPCR assay is highly reliable due to plasmid-normalization, use of standard curves, and a high level of standardization in the experimental assembly. Additionally, direct comparison of array based mtDNA-CN shows more pronounced associations with traits (mean ß-effect is 5.8 times higher) than qPCR-measured mtDNA-CN^74^.

This project has numerous other advantages: all mtDNA-CN measurements were conducted in a standardized way in the same laboratory guaranteeing a consistant high quality. We handled samples of all studies uniformly, measured in triplicate and performed the standardized assay with plasmid normalization and included two additional reference DNA samples on each plate to control the inter-assay variability. Furthermore, we were able to apply various models with different covariate adjustment including smoking and/or blood cell counts into the model. By this, we were able to dissect whether a genetic variant’s association with mtDNA-CN was mitigated by blood cell count and additionally, this enabled the precise determination of the impact each covariate has on the identified variants. Finally, the highly automated nf-gwas pipeline ensured validation of input data and further quality control^32^. The pipeline enhances the reproducibility of the analysis, limiting the influence of error prone procedures in QC and genetic data preparation. All steps are controlled through a configuration file where the analysis parameters are defined, avoiding custom scripting for each step. Limitations of the study include the much smaller sample size compared to the recently published GWAS (e.g. Longchamps et al.^15^ with UKB data). Nevertheless, to our knowledge our study is so far the largest GWAS on qPCR-measured mtDNA-CN. Unfortunately, blood cell counts were only available for 11,438 participants from the CHRIS and AugUR studies (~ 70% of total sample size). An issue for investigating mt variants is the use of different genotyping arrays. The five platforms cover different SNPs and therefore the overlap of available mitochondrial SNPs over the entire studies was not ideal, however, was improved by imputation via MitoImpute*.*

## Conclusion

Our meta-analysis of 16,130 individuals revealed two significant loci associated with mtDNA-CN based on the main model adjusted for age, sex and 4 PCs. We did not find major differences, as equivalence tests between our results and the largest available GWAS only highlighted two SNPs as not being equivalent. Since we only identified one rare mitochondrial variant, we believe this demonstrates that the mitochondrial genome itself contributes only marginally to mtDNA-CN regulation.

### Supplementary Information

Supplementary Information.

Supplementary Tables.

## Data Availability

GWAS summary statistics of the main model are available at the address: https://genepi.i-med.ac.at/data/mtdna-cn-meta-gwas/. Further summary statistics and datasets generated and/or analyzed during within the project at hand are available from the corresponding author on reasonable request and after approval of the involved studies.
