# Supplementary material for: Nuclear and mitochondrial genetic variants associated with mitochondrial DNA copy number
Source: Sci Rep. 2024 Jan 24;14:2083. doi: 10.1038/s41598-024-52373-0 (PMC10808213; doi:10.1038/s41598-024-52373-0)
Supplement: Supplementary file 1 — Supplementary Information. [file 41598_2024_52373_MOESM1_ESM.pdf]

Supplemental Material for

Nuclear and mitochondrial genetic variants associated with mitochondrial DNA copy number

Adriana Koller[1†], Michele Filosi[2†], Hansi Weissensteiner[1†], Federica Fazzini[1], Mathias Gorski[3], Cristian Pattaro[2], Sebastian Schönherr[1], Lukas Forer[1], Janina M Herold[3], Klaus J Stark[3], Patricia Döttelmayer[1], Andrew A. Hicks[2], Peter P. Pramstaller[2], Reinhard Würzner[4], Kai-Uwe Eckardt[5,6], Iris M Heid[3], Christian Fuchsberger[2#], Claudia Lamina[1#], Florian Kronenberg[1#*]

[1] Institute of Genetic Epidemiology, Medical University of Innsbruck, Innsbruck, Austria

[2] Eurac Research, Institute for Biomedicine, Affiliated Institute of the University of Lübeck, Bolzano, Italy

[3] Department of Genetic Epidemiology, University of Regensburg, Regensburg, Germany

[4] Institute of Hygiene and Medical Microbiology, Medical University of Innsbruck, Innsbruck, Austria

[5] Department of Nephrology and Hypertension, University Hospital Erlangen, Friedrich-Alexander-Universität Erlangen-Nürnberg, Erlangen, Germany and German Chronic Kidney Disease study

[6] Department of Nephrology and Medical Intensive Care, Charité – Universitätsmedizin Berlin, Berlin, Germany

†These authors contributed equally.
[#] These authors contributed equally.
* Corresponding author

Corresponding author:
Florian Kronenberg, MD

Institute of Genetic Epidemiology
Medical University of Innsbruck
Schöpfstrasse 41, A-6020 Innsbruck, Austria.
Phone: (+43) 512-9003-70560
Fax: (+43) 512 9003-73560 or -73561,
Email: florian.kronenberg@i-med.ac.at

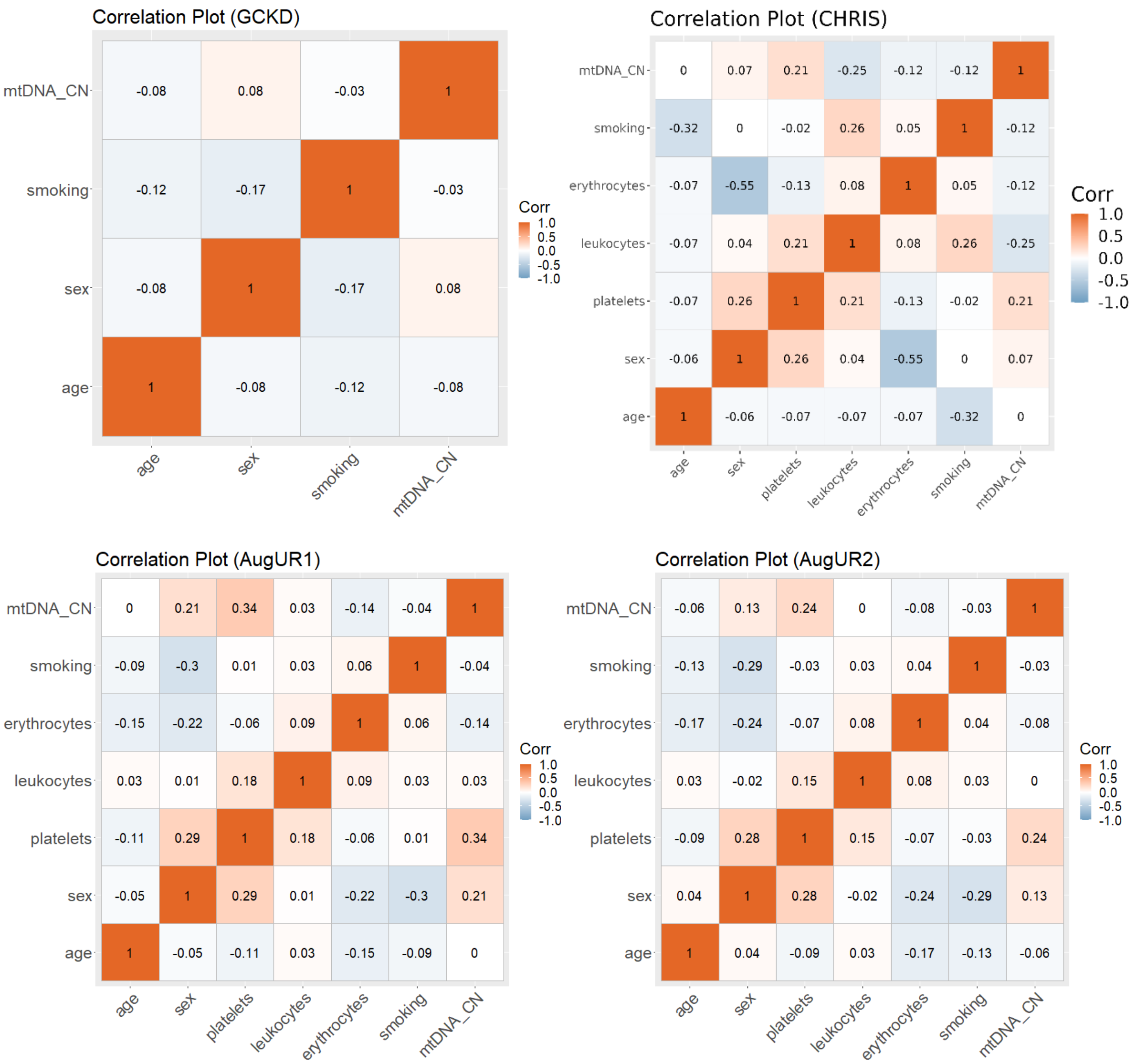

Figure S1: Correlation plot (Pearson correlation coefficient) to test linear relation between mtDNA-CN and covariates in each study. Red indicates a positive correlation, while blue indicate negative correlation coefficients.

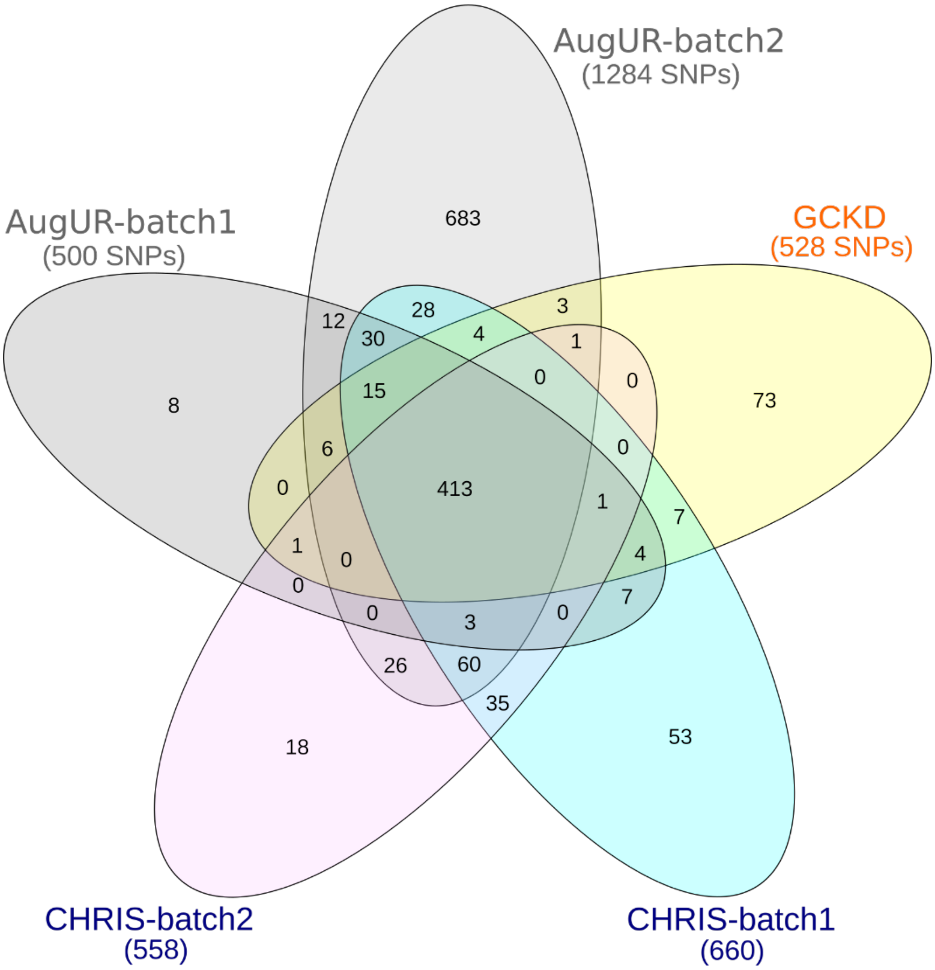

Figure S2: Venn Diagram illustrating differences in genotyped mitochondrial variants on the five used microarrays. In CHRIS, the Illumina OmniExpressExome and the OMNI 2.5Exome chip array, in the GCKD study, the OMNI 2.5Exome BeadChip and in the AugUR study, different versions of the Illumina Global Screening Array (v1/v3) were used for genotyping.

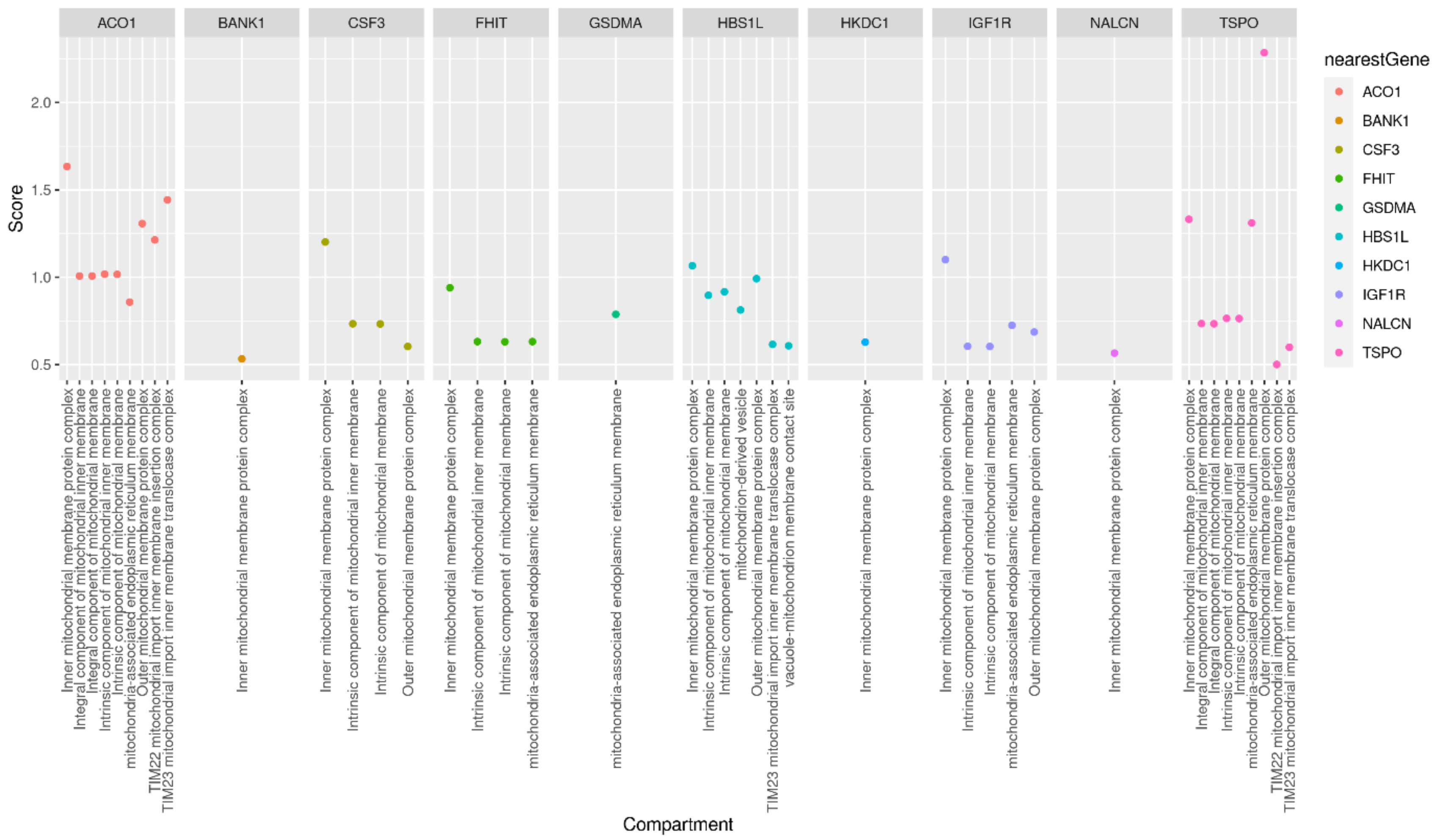

Figure S3: Mitochondrial sub-compartments were analyzed for all genes with COMPARTMENTS resource [1], integrating all sources of protein subcellular localization information (including manually curated literature, high-throughput microscopy-based screens, primary sequence predictions, and automatic text mining results), based on the meta-analysis top hits. Scores from https://download.jensenlab.org/human_compartment_integrated_full.tsv ranking between 0.5 and 5 (highest confidence) limited to mitochondria related compartments.

A

Manhattan Plot adjusted for age, sex, smoking, 4 PCs

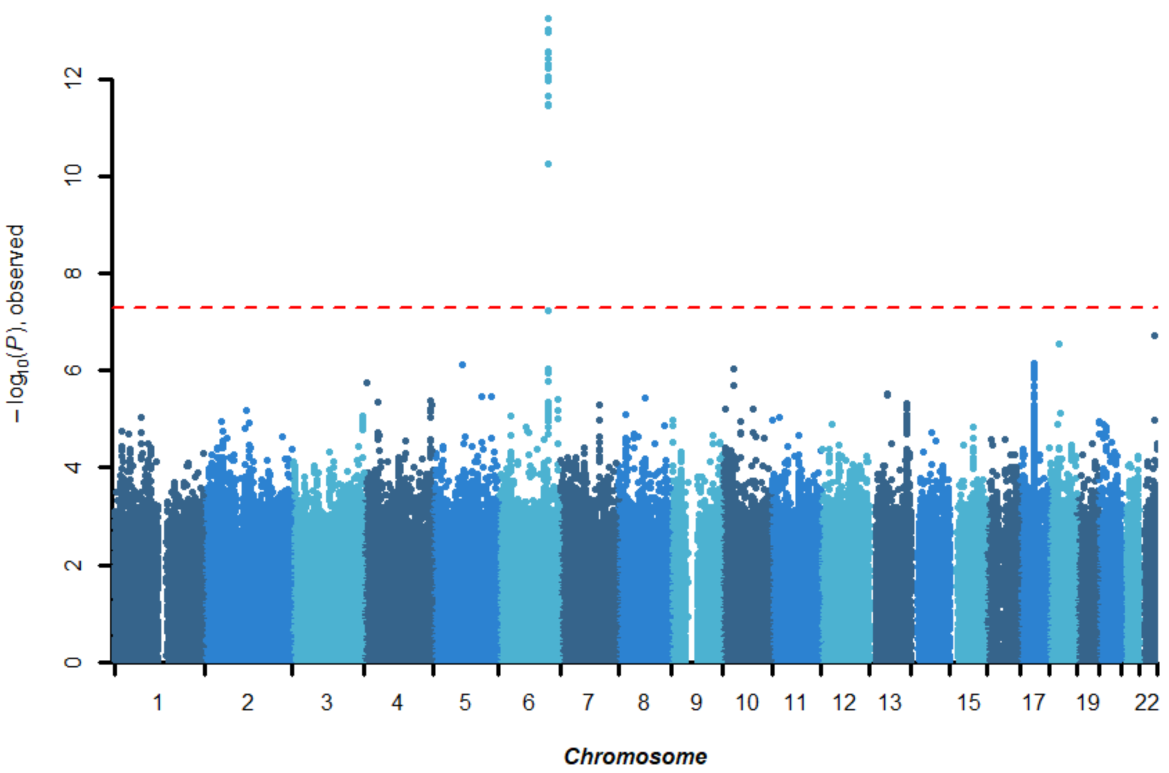

B

Manhattan Plot adjusted for age, sex, RBC, 4 PCs

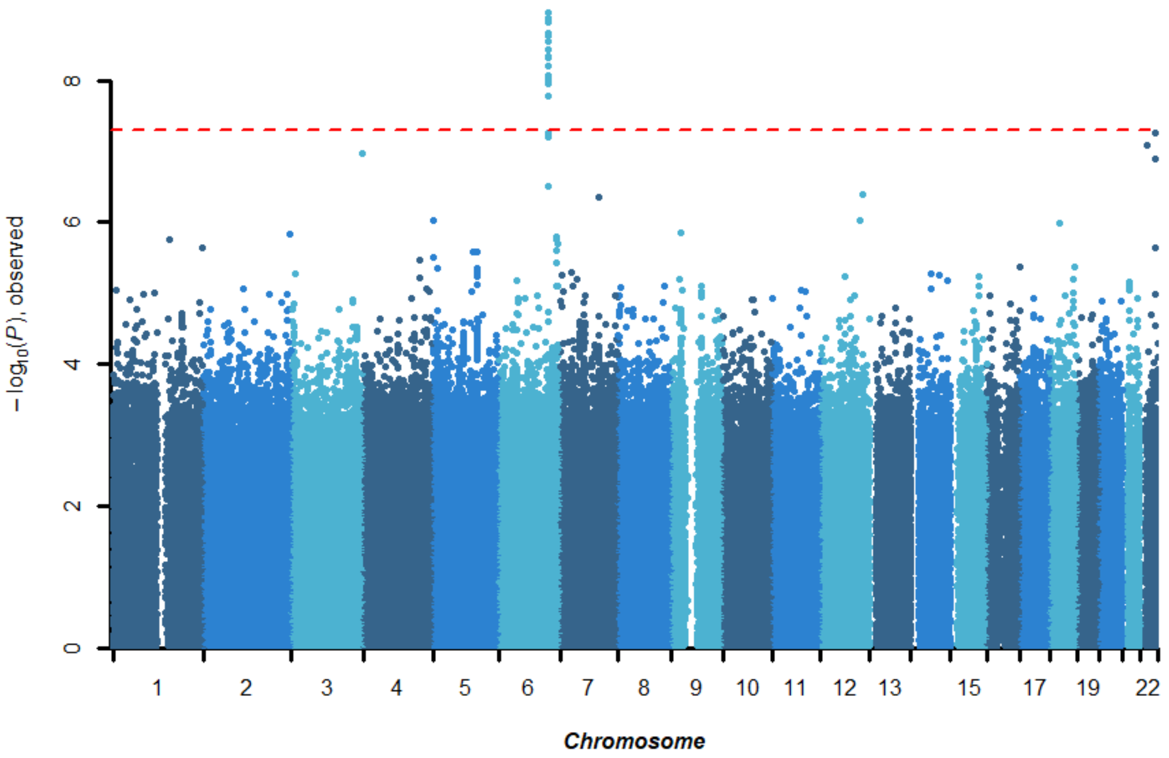

C

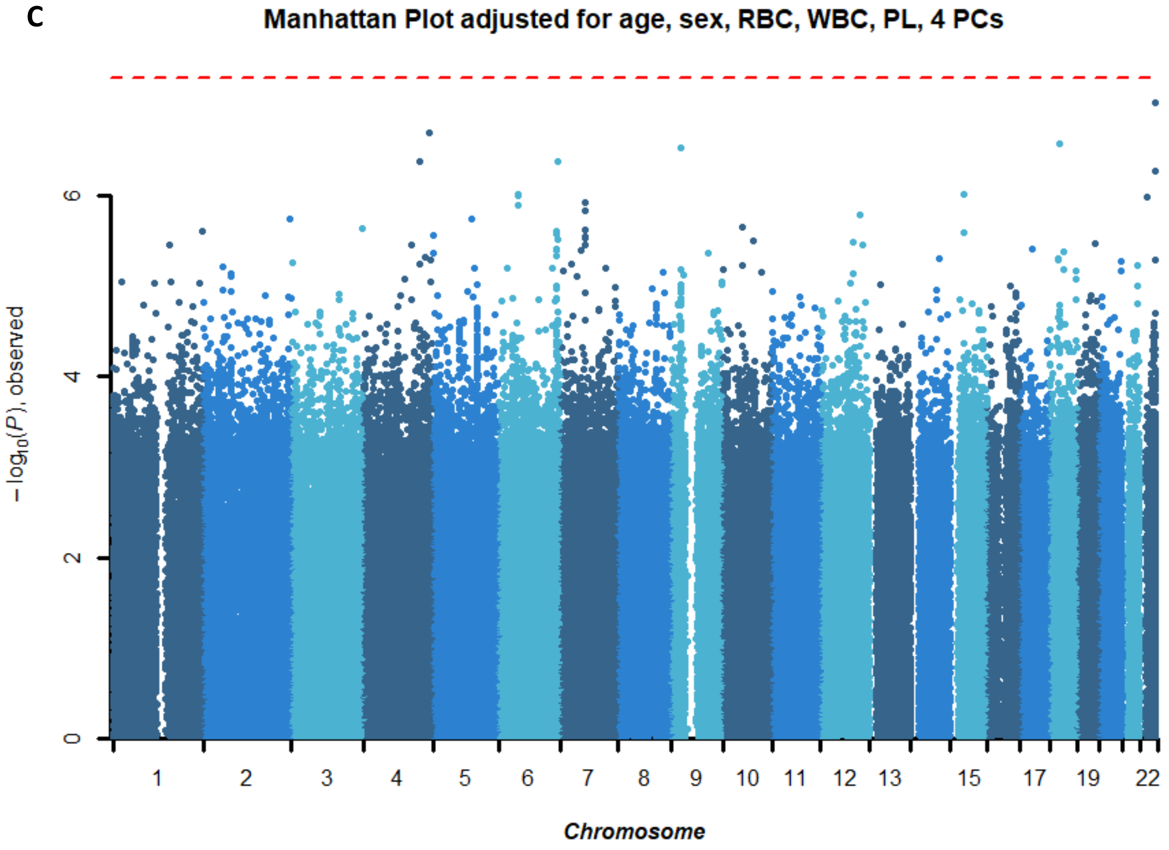
Manhattan Plot adjusted for age, sex, RBC, WBC, PL, 4 PCs
$-\log_{10}(P)$, observed
0
2
4
6
1 2 3 4 5 6 7 8 9 10 11 12 13 15 17 19 22
Chromosome

D

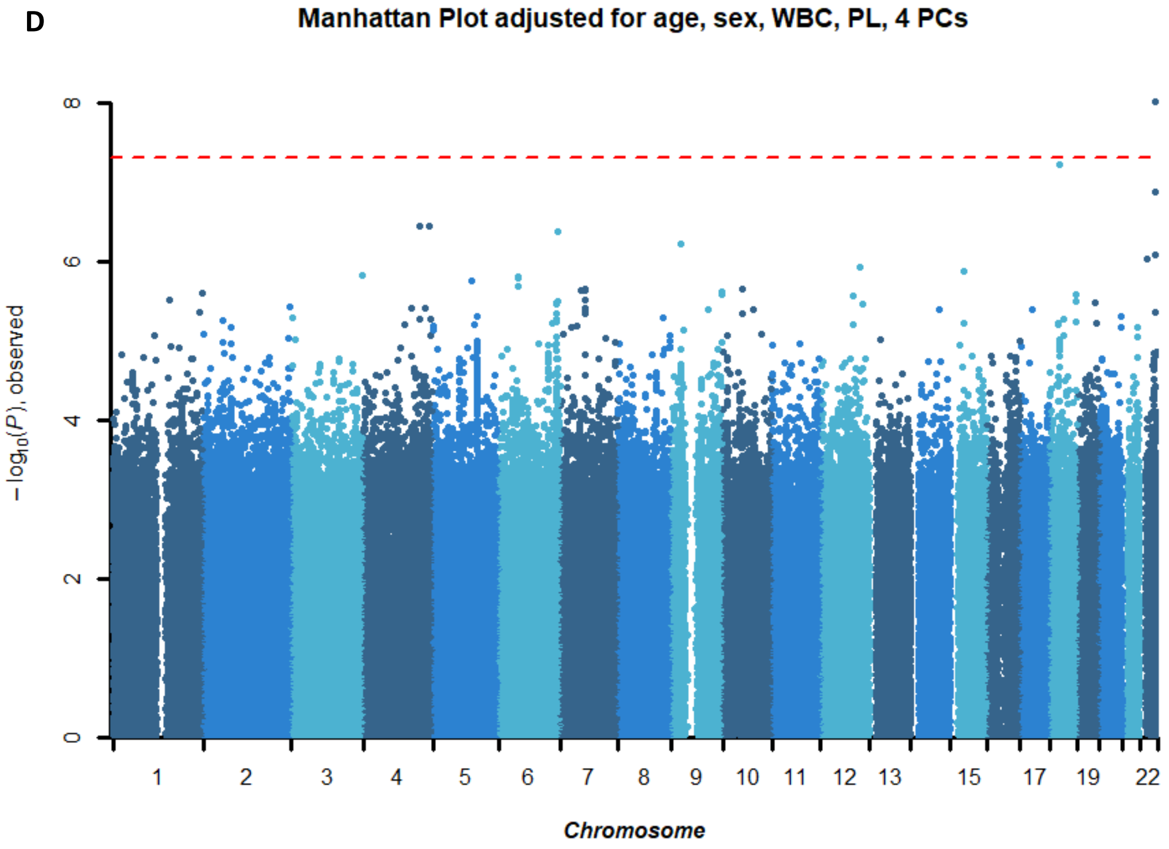
Manhattan Plot adjusted for age, sex, WBC, PL, 4 PCs
$-\log_{10}(P)$, observed
0
2
4
6
8
1 2 3 4 5 6 7 8 9 10 11 12 13 15 17 19 22
Chromosome

E

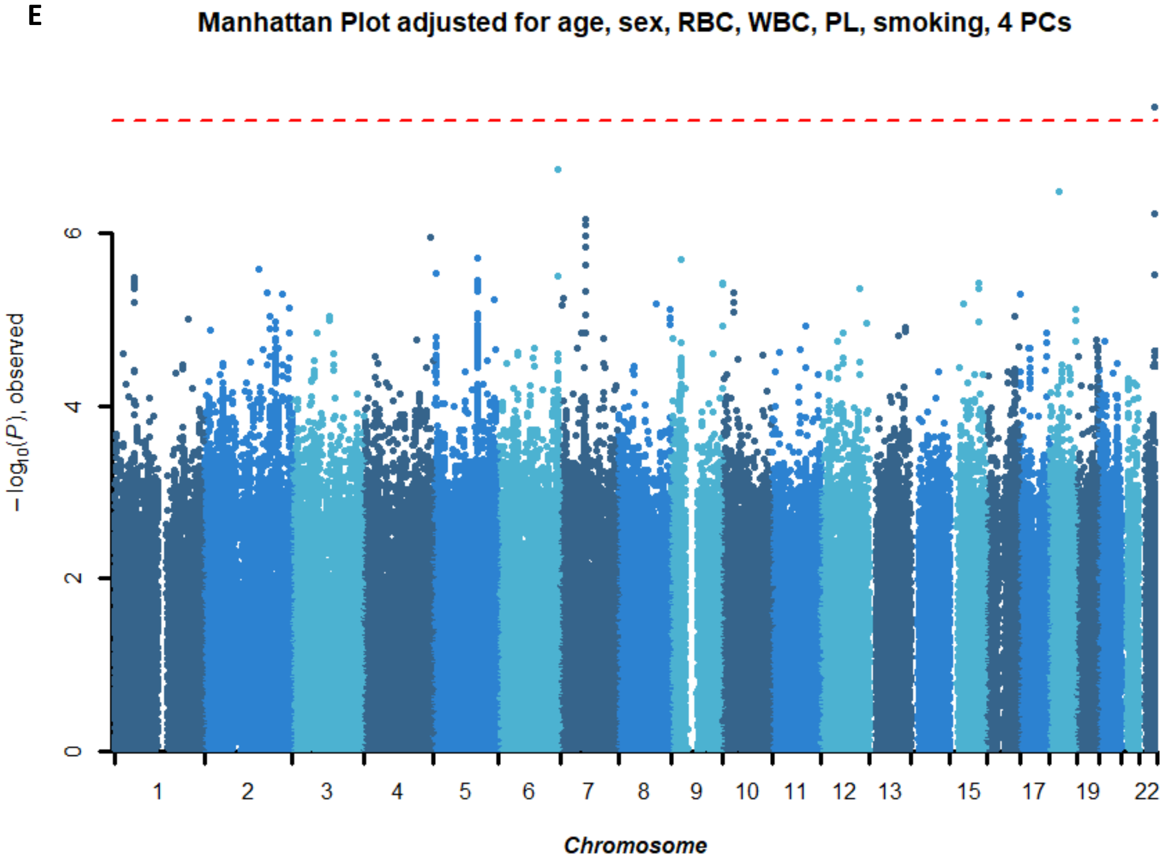

Figure S4: Manhattan plots illustrating meta-analyses of genome-wide association studies for mtDNA-CN from all three studies (GCKD, AugUR, CHRIS) for all additional adjustment models (A-E). The red line represents the threshold for genome-wide significance (p-value<5×10^{-8}). The x-axis gives the chromosomes, the y-axis shows the –log10 p-values of imputed SNPs. Abbreviations: RBC = red blood cell count, WBC = white blood cell count, PL = platelets count, PC = principal components.

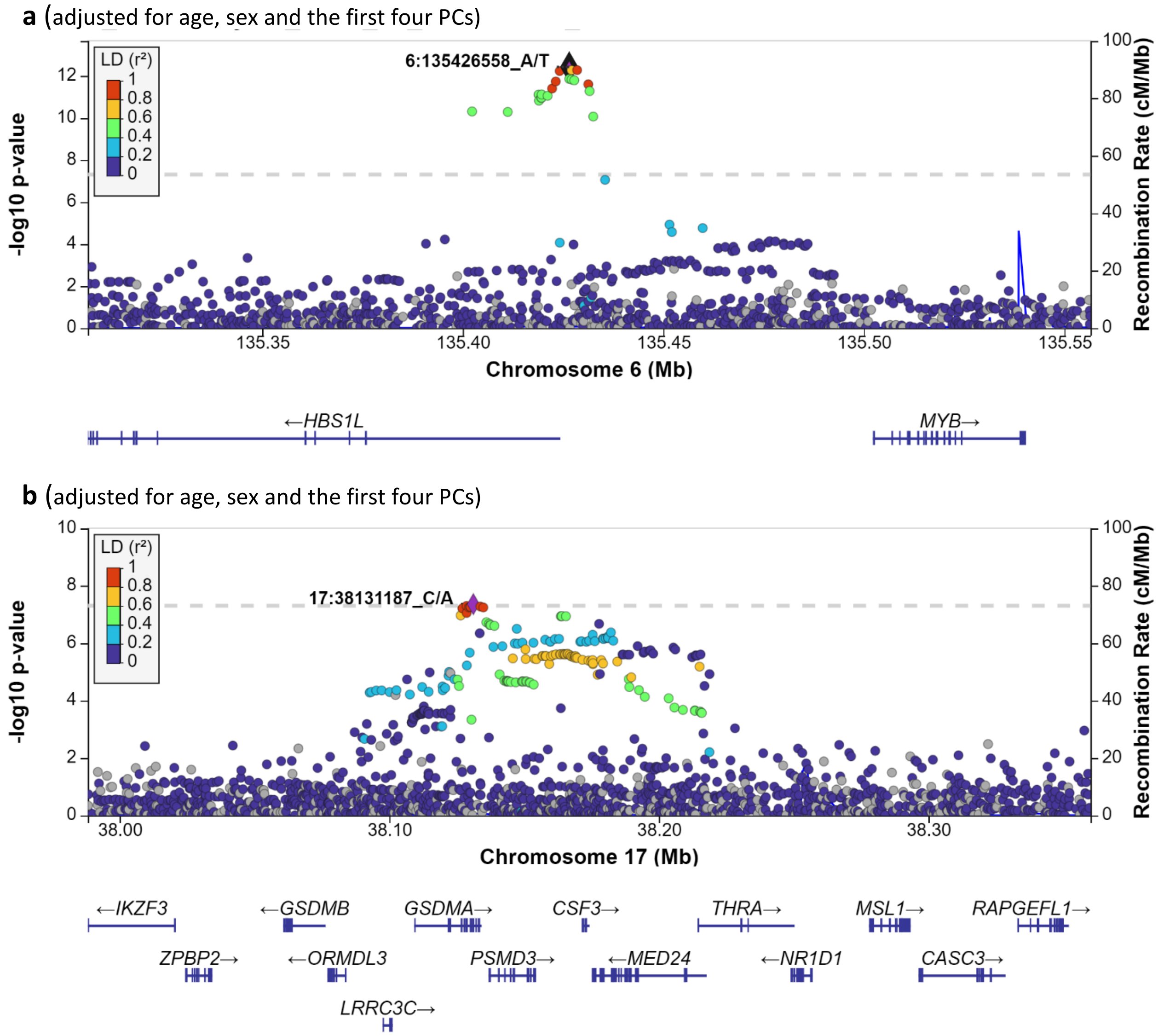

Figure S5: Regional plots created with LocalZoom [2] showing a detailed picture of the genome-wide significant loci. The two regions associated with mtDNA-CN are shown: (a) the variant between *HBS1L* and the *MYB* gene, (b) the missense variant in the *GSDMA* gene. (a) and (b) show the main model adjusted for age, sex and the first four PCs. The linkage disequilibrium (LD) of each variant with the labelled lead SNP is indicated by color. The x-axis gives the chromosome position and below the genes within the plotted genomic region. The y-axes show the –log10 p-values of imputed SNPs as well as the recombination rate.

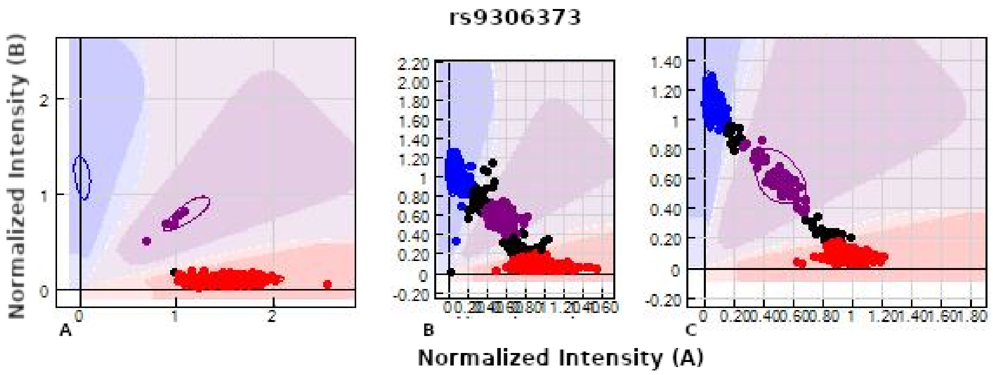

Figure S6: Cluster plot for variant rs9306373 (chromosome 22, genomic position 43560682) from the Illumina GenomeStudio software for batches 1, 2 and 3 in the CHRIS dataset in panel A, B and C respectively. For the specific variant the Illumina calling algorithm show poor performances for batches 2 and 3 (panel B and C respectively) with a high number of calls falling outside the defined cluster thresholds.

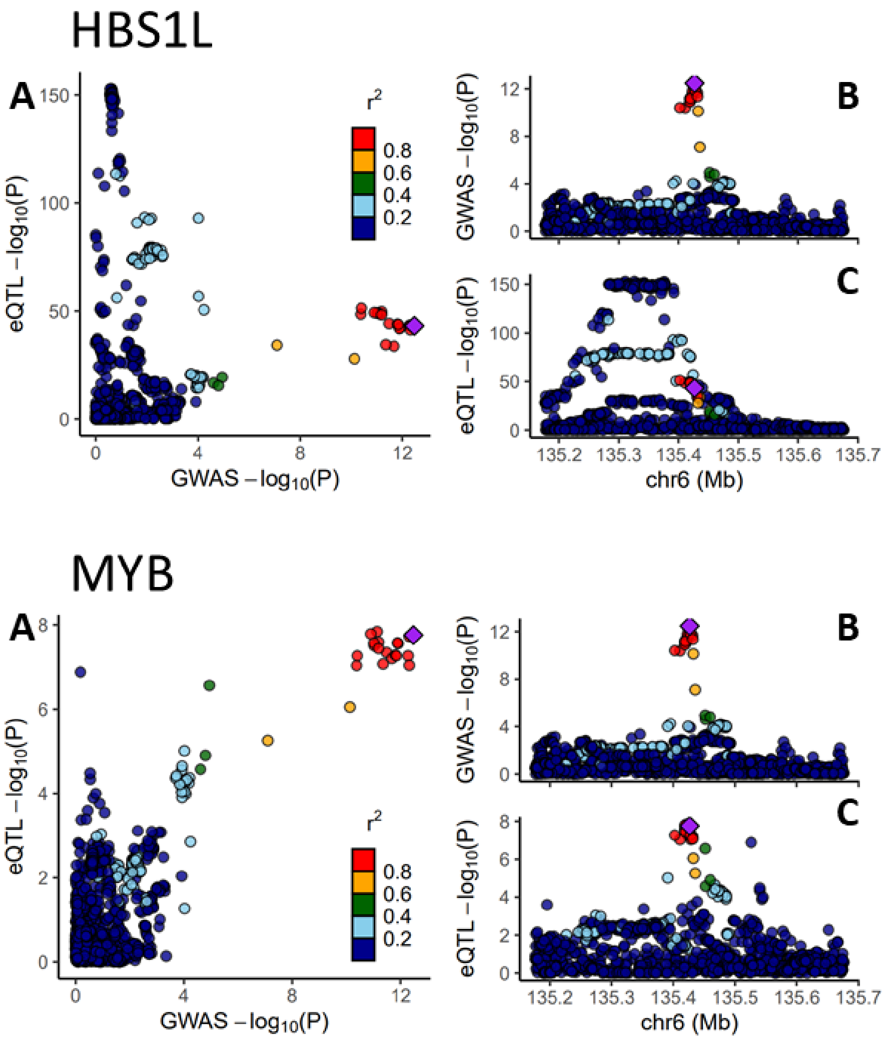

Figure S7: Results of colocalization analysis of HBS1L and MYB in whole blood. The purple square represents our GWAS top hit (rs4895440) on chromosome 6 in all plots. Results are shown for all SNPs in the region ±250kB surrounding rs4895440. For each gene, (A) shows the colocalization of mtDNA-CN GWAS and eQTL signals. (B) shows the p-values from our mtDNA-CN GWAS (C) shows the p-values of the association on the expression levels on *HBS1L* respectively *MYB* obtained from the eQTLGen Consortium [3].

GSDMA

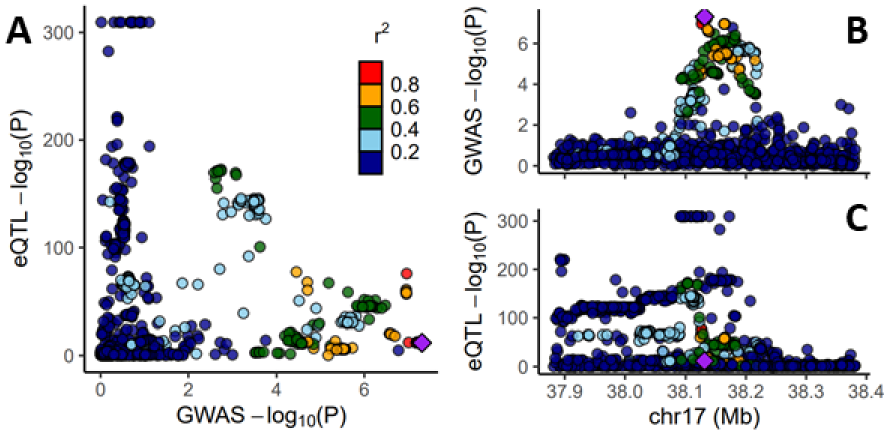

GSDMB

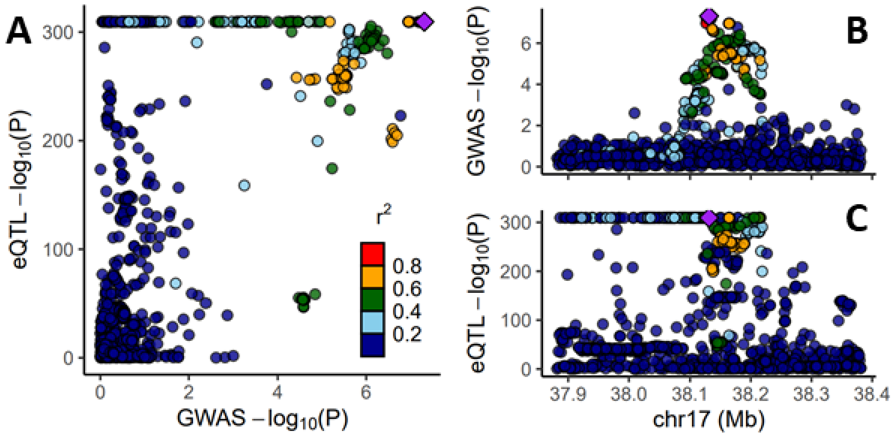

IKZF3

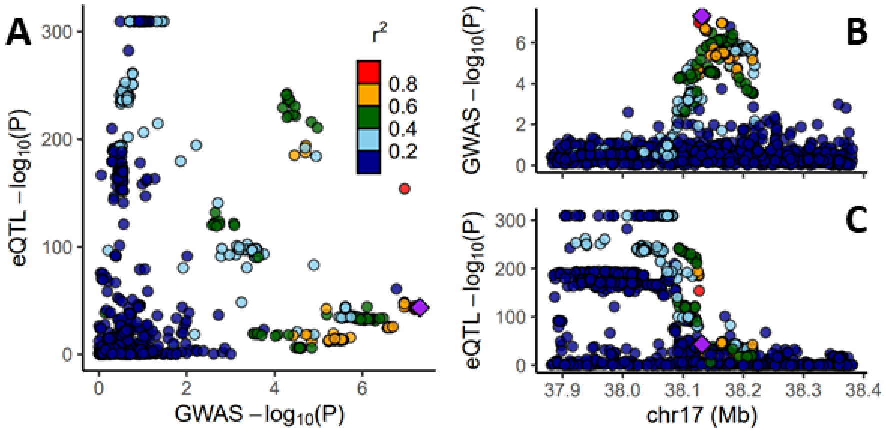

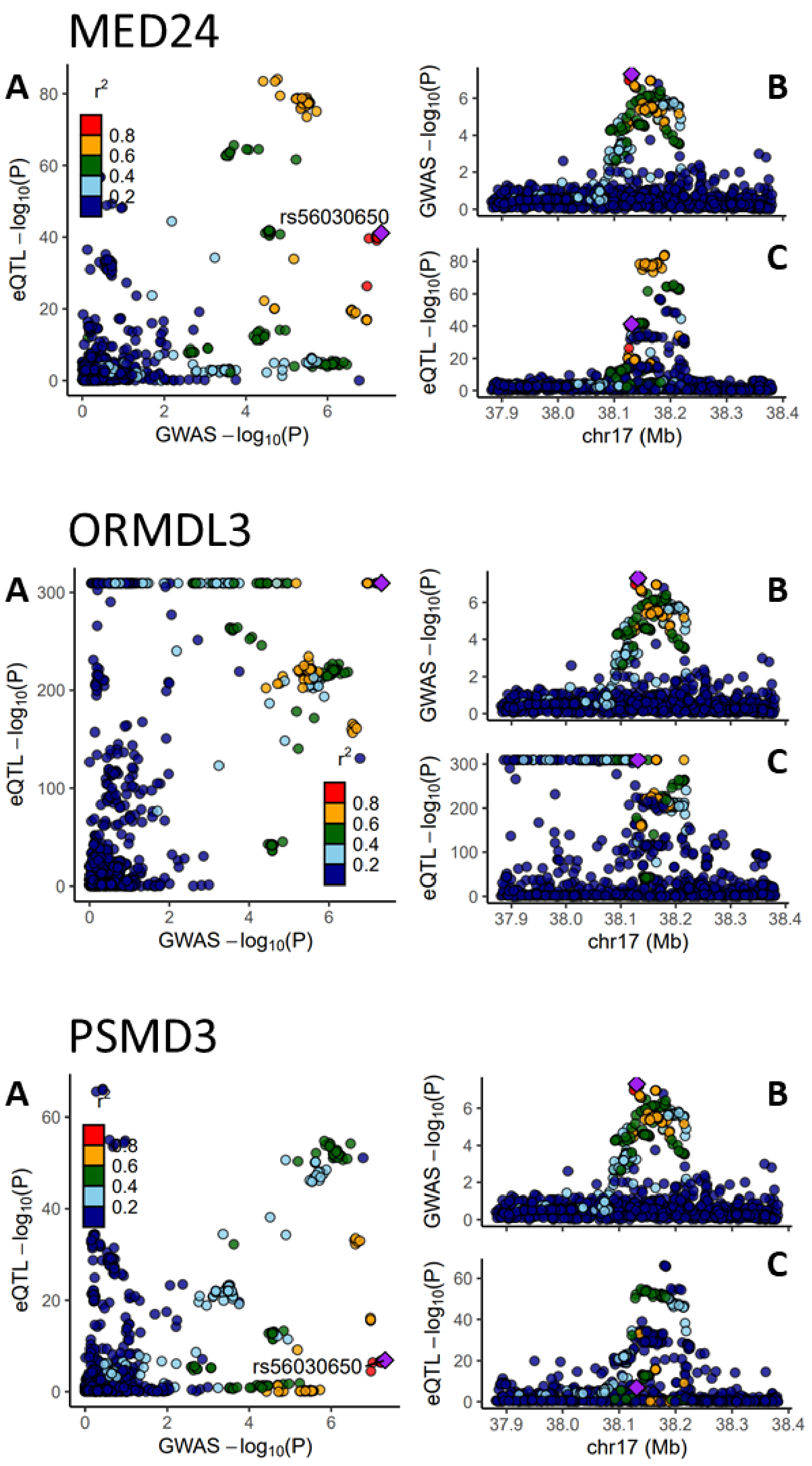

Figure S8: Results of colocalization analysis of genes around our GWAS lead SNP on chromosome 17 in whole blood. The purple square represents our GWAS top hit (rs56030650) in all plots. Results are shown for all SNPs in the region ±250kB surrounding rs56030650. For each gene, (A) shows the colocalization of mtDNA-CN GWAS and eQTL signals. (B) shows the p-values from our mtDNA-CN GWAS (C) shows the p-values of the association on the expression levels on the respective gene obtained from the eQTLGen Consortium [3].

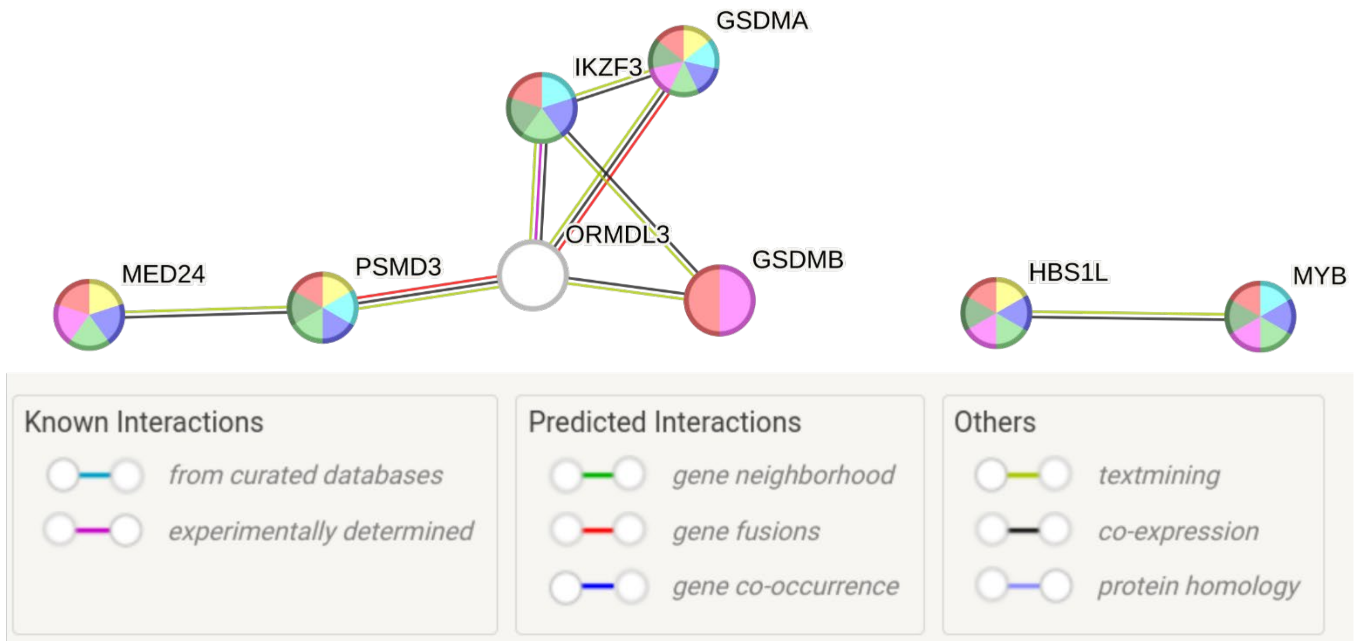

Human Phenotype (Monarch)

phenotype	description	count in network	strength	false discovery rate
EFO:0007614	Asthma exacerbation measurement	3 of 33	2.35	0.00048
EFO:0006312	Mitochondrial DNA measurement	4 of 82	2.08	0.00028
EFO:0010638	Atopic asthma	4 of 88	2.05	0.00028
EFO:0007993	Lymphocyte percentage of leukocytes	4 of 320	1.49	0.0036
EFO:0004833	Neutrophil count	6 of 712	1.32	0.00028
EFO:0004842	Eosinophil count	6 of 734	1.3	0.00028
EFO:0005091	Monocyte count	5 of 639	1.28	0.0019
EFO:0004587	Lymphocyte count	5 of 694	1.25	0.0025
EFO:0004582	Liver enzyme measurement	5 of 1124	1.04	0.0190
EFO:0004308	Leukocyte count	7 of 2057	0.92	0.0011

Figure S9: Protein-protein interaction network analysis using the STRING database, based on the findings of our colocalization analysis. The eight nodes in the network fall into two clusters, with high interaction (PPI enrichment p-value: 1.75×10^{-09}). The node colors indicate connections with phenotypes curated by the Monarch Initiative [4], with the highlighted entries in the list considered.

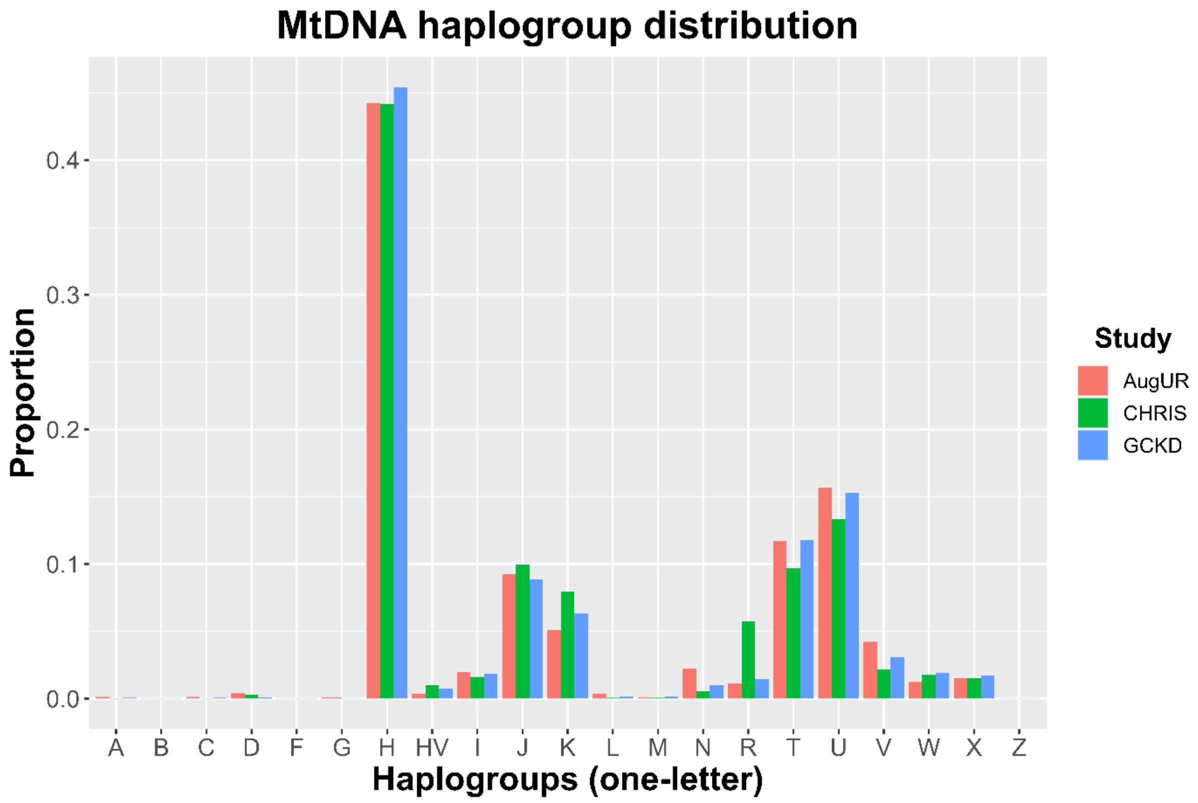

Figure S10: Distribution of haplogroups separated for each study cohort. Haplogroups and macrohaplogroups are shown on the x-axis, the frequency is represented on the y-axis. MitoImpute [5] with the Reference Panel v1 0.01 (MAF 1%) was used to infer missing mtDNA variants and final haplogroup estimation using HaploGrep 2 (version 2.4) [6] was performed in 16,121 samples.

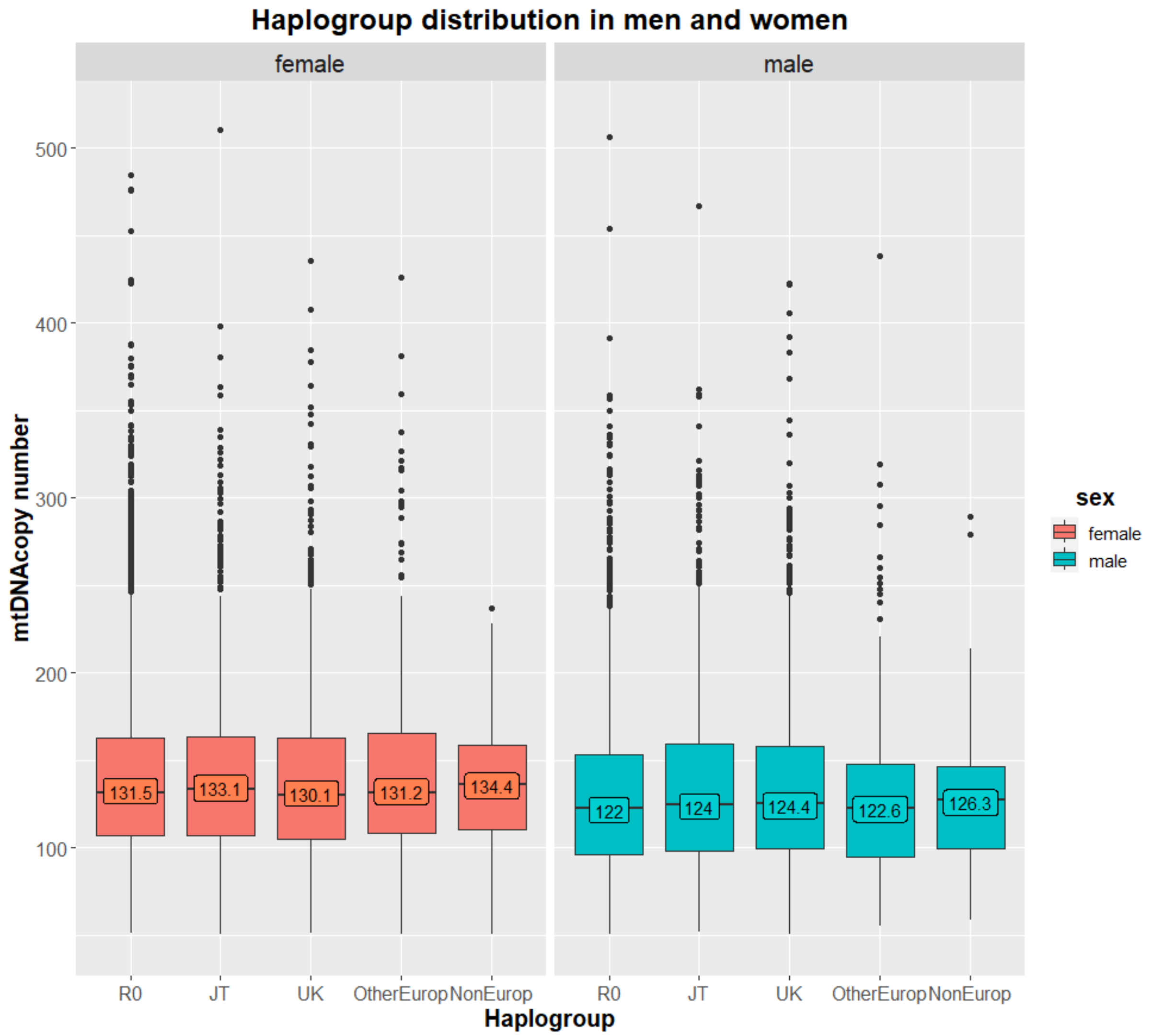

Figure S11: Mitochondrial DNA copy number distribution between the five haplogroup-clusters (R0, JT, UK, Other Europeans, Non-Europeans) for men and women separately. Medians of mitochondrial DNA copy number for each cluster are labelled.

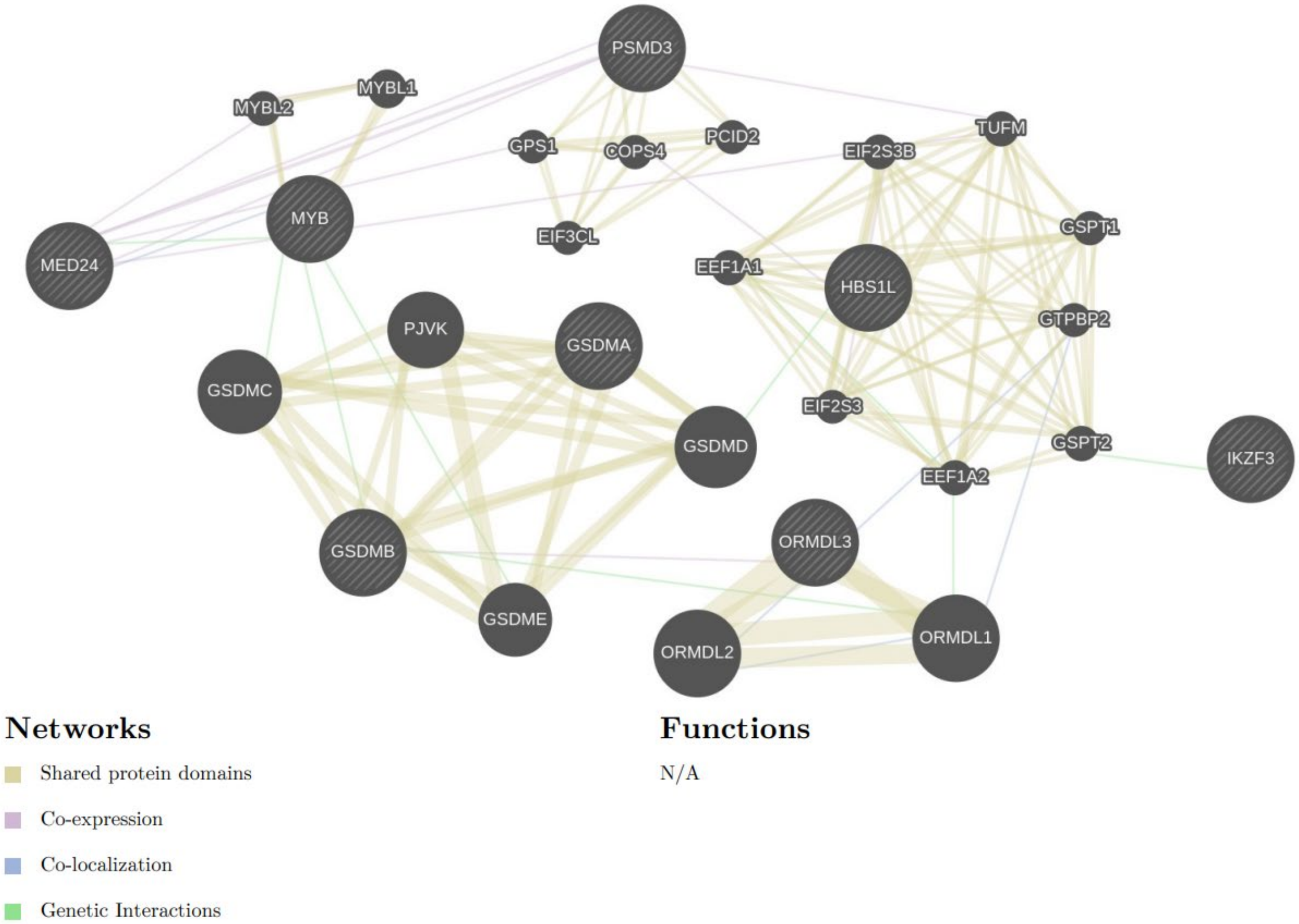

Figure S12: Protein network interaction exploration using GenMANIA [7]. Input genes were chosen based on genes identifies in colocalization analysis (nodes with dashed background).

Table S1: Overview on previous studies investigating genetic aspects of mtDNA copy number regulation from blood samples.

Study (Journal)	Sample Size	Method to determine mtDNA-CN	Method (genotyping)	Investigation of mitochondrial variants (1) or haplogroups (2)	Considering blood cell counts	Main Results	Additional Information
Curran JE et al., 2007 (Hum Mol Genet) [8]	N=1,259	qPCR	Short tandem repeat markers using MapPairs version 6, SOLAR	No/No	No	829 transcripts influencing mt content; 1 autosomal QTL and 1 mt QTL	Estimation of the heritability of mt content
López S et al., 2014 (Mitochondrion) [9]	N=386	qPCR	Illumina Infinium 317K Beadchip	No/No	No	Two intron-polymorphisms; sex-specific control of mtDNA levels	
Cai N et al., 2015 (Current Biology) [10]	N=10,442	WGS	low coverage sequencing Illumina HiSeq 2000 (1.7 x nDNA, 102x mtDNA)	Yes/Yes	No	Two loci	
Workalemahu T et al., 2017 (J Matern Fetal Neonatal Med) [11]	N=471	qPCR	Illumina Cardio-Metabo Chip	No/No	No	No genome-wide significant hit	
Li Z et al., 2018 (BMC Genomics) [12]	N=301	qPCR	Illumina Human Exome BeadChip	No/No	No	Seven SNPs and 11 genes associated with mtDNA-CN	
Guyatt AL et al., 2019 (Human Genomics) [13]	N=6,799/ N=11,253	qPCR / meta-an.	Illumina Human660W-Quad array / HumanHap550-Quad array / 1.2 M Duo platform	No/No	Yes	one locus in females, lower signal for TFAM	
Ganel L et al., 2021 (Human Genomics) [14]	N=19,184	WGS, WES	WGS / WES / Illumina 610-Quad SNP array	No/Yes	Yes	Two loci	
Hägg S et al., 2021 (Human Genetics) [15]	N=295,150 (UKB)	WES, array based	Affymetrix arrays	No/Yes	Yes	50 independent regions	
Gentiluomo M et al., 2021 (Mitochondrion) [16]	N=301,986 (UKB, ESTHER)	qPCR	Illumina Infinium OncoArray / Global Screening Array BeadChips	No/No	Yes	Two novel loci	“mitoscore” (polygenic score incl. known variants)
Longchamps RJ et al., 2022 (Human Genetics) [17]	N=465,809 (UKB, CHARGE)	qPCR/WGS/ WES/array based	Different versions of Illumina arrays, Affymetrix 6.0, Affymetrix UK Biobank Axiom/ Affymetrix UK BiLEVE Axiom array	Yes/Yes	Yes	129 autosomal SNPs (87 independent across 96 loci), 4 X-Chr. SNPs	MitoPipeline; investigated X Chromosome
Chong M et al., 2022 (eLife) [18]	N=395,781	WES/qPCR/array based	Axiom Precision Medicine Research Array, Affymetrix UK Biobank Axiom/ Affymetrix UK BiLEVE Axiom array	No/No	Yes	73 loci	AutoMitoC; investigated X Chromosome
Pillalamarri V et al., 2022 (HGG Adv) [19]	N=415,422 (UKB)	WES	Affymetrix UK Biobank Axiom/ Affymetrix UK BiLEVE Axiom array	No/Yes (covariates)	Yes	24 independent signals from 17 loci	
Zaidi AA et al., 2023 (HGG Adv) [20]	N=30,666	WGS	Illumina Infinium Global Screening Array	No/Yes	Yes	No genome-wide significant hit	Investigated different ancestries
Gupta R et al., 2023 (Nature) [21]	N=274,832 (UKB, AoU)	WGS	WGS / Affymetrix UK Biobank Axiom/ Affymetrix UK BiLEVE Axiom array	No/Yes	Yes	92 nuclear loci	MtSwirl pipeline, investigated mtDNA heterioplasmy
Koller A et al., (this study)	N=16,130	qPCR	Illumina Human OmniExpressExon, OMNI 2.5Exome, OMNI 2.5Exome BeadChip and Infinium® Global Screening Array v1/v3	Yes/Yes	Yes	2 nuclear and 1 mt variant	

Abbreviations: QTL = quantitative trait loci, WGS = whole genome sequencing, WES = whole exome sequencing, qPCR = quantitative PCR, SNP = single nucleotide polymorphism, GWAS = genome-wide association studies, UKB = UK Biobank, nuc = nuclear, mt = mitochondrial, AoU = AllofUs

Table S2: Overview on ß-estimates and p-values of three top hits in all different adjustment models including an additional main model without GCKD to allow differentiation between power effects and influence on different adjustment variables.

Statistical model	Included studies	ß (95%CI)	p-value	N
**Chr. 6:135426558 (between *HBS1L* and *MYB* genes)**				
Main model (age, sex, 4 PCs)	CHRIS, GCKD, AugUR	0.086 (0.06; 0.11)	$3.39x10^{-13}$	16130
Main model (age, sex, 4 PCs) without GCKD	CHRIS, AugUR	0.092 (0.06; 0.12)	$4.60x10^{-11}$	11438
Model 2 (age, sex, smoking, 4 PCs)	CHRIS, GCKD, AugUR	0.089 (0.07; 0.11)	$5.64x10^{-14}$	16052
Model 3 (age, sex, RBC, 4 PCs)	CHRIS, AugUR	0.086 (0.06; 0.11)	$1.33x10^{-09}$	11333
Model 4 (age, sex, RBC, WBC, PLT, 4 PCs)	CHRIS, AugUR	0.050 (0.02; 0.08)	$1.90x10^{-04}$	11333
Model 5 (age, sex, WBC, PLT, 4 PCs)	CHRIS, AugUR	0.058 (0.03; 0.08)	$1.43x10^{-05}$	11333
Model 6 (age, sex, RBC, WBC, PLT, smoking, 4 PCs)	CHRIS, AugUR	0.048 (0.02; 0.07)	$4.23x10^{-04}$	11269
**Chr. 17:38131187 (*GSDMA*)**				
Main model (age, sex, 4 PCs)	CHRIS, GCKD, AugUR	-0.057 (-0.08; -0.04)	$4.85x10^{-08}$	16130
Main model (age, sex, 4 PCs) without GCKD	CHRIS, AugUR	-0.052 (-0.08; -0.03)	$2.77x10^{-05}$	11438
Model 2 (age, sex, smoking, 4 PCs)	CHRIS, GCKD, AugUR	-0.053 (-0.07; -0.03)	$7.10x10^{-07}$	16052
Model 3 (age, sex, RBC, 4 PCs)	CHRIS, AugUR	-0.050 (-0.07; -0.03)	$5.95x10^{-05}$	11333
Model 4 (age, sex, RBC, WBC, PLT, 4 PCs)	CHRIS, AugUR	-0.031 (-0.05; -0.01)	$8.39x10^{-03}$	11333
Model 5 (age, sex, WBC, PLT, 4 PCs)	CHRIS, AugUR	-0.033 (-0.06; -0.01)	$6.00x10^{-03}$	11333
Model 6 (age, sex, RBC, WBC, PLT, smoking, 4 PCs)	CHRIS, AugUR	-0.029 (-0.05; -0.01)	$1.63x10^{-02}$	11269

Table S3: Allele frequencies and number of carriers of top hits for the main model (adjusted for age, sex, 4 PCs) for each study separately.

	Study	Chr	Position	Allele0	Allele1	A1Freq	Info	N	Effect	SE	p-value	N Carrier A1	N Carrier A0
**rs4895440**	GCKD	6	135426558	A	T	0.287	0.997	4692	0.069	0.021	$1.34x10^{-03}$	1347	3345
	AugUR1	6	135426558	A	T	0.276	0.976	956	0.069	0.042	$1.03x10^{-01}$	264	692
	AugUR2	6	135426558	A	T	0.258	0.995	1162	0.141	0.043	$1.06x10^{-03}$	299	863
	CHRIS	6	135426558	A	T	0.259	0.996	9320	0.089	0.016	$1.73x10^{-08}$	2414	6906
**rs56030650**	GCKD	17	38131187	C	A	0.449	1.009	4692	-0.070	0.019	$3.19x10^{-04}$	2108	2584
	AugUR1	17	38131187	C	A	0.431	0.972	956	-0.043	0.038	$2.60x10^{-01}$	412	544
	AugUR2	17	38131187	C	A	0.428	1.003	1162	-0.083	0.038	$2.92x10^{-02}$	497	665
	CHRIS	17	38131187	C	A	0.421	0.985	9320	-0.049	0.014	$4.99x10^{-04}$	3921	5399
**rs9306373***	GCKD	22	43560682	A	G	0.005	1.029	4692	0.265	0.136	$5.14x10^{-02}$	23	4669
	AugUR1	22	43560682	A	G	0.004	0.879	956	0.016	0.300	$9.57x10^{-01}$	4	952
	AugUR2	22	43560682	A	G	0.005	0.951	1162	0.321	0.279	$2.48x10^{-01}$	6	1156
	CHRIS	22	43560682	A	G	0.070	0.929	9320	-0.169	0.028	$2.79x10^{-09}$	655	8665

Abbreviations: Chr = Chromosome; Allele1 = effect allele; SE = standard deviation of ß-estimate (effect)

* This variant was considered a technical artifact and therefore, excluded from further considerations.

Table S4: Results of eQTL and colocalization analysis in whole blood using data from the eQTLGen Consortium (n=31,684) [3]

	eQTL data of the respective gene			LD of eQTL lead SNP with GWAS lead SNP[c]		Posterior probability for the hypothesis	
**Genes[a]**	***p*-value of GWAS lead SNP / FDR**	**eQTL Lead SNP**	***p*-value of eQTL lead SNP / FDR[b]**	**D'**	**r^2**	**H3**	**H4**
**Chromosome 6 gene region with GWAS lead SNP rs4895440**							
HBS1L	6.21×10^{-44} / 0	rs12526055	1.54×10^{-153} / 0	0.66	0.17	1.00	<0.001
MYB	1.74×10^{-8} / 9.61×10^{-5}	rs34164109	1.44×10^{-8} / 7.06×10^{-5}	0.98	0.90	0.02	0.98
**Chromosome 17 gene region with GWAS lead SNP rs56030650**							
IKZF3	3.80×10^{-44} / 0	rs2941519	3.27×10^{-310} / 0	0.43	0.16	1.00	<0.001
GSDMB	3.27×10^{-310} / 0	GWAS lead SNP		--	--	0.24	0.76
ORMDL3	3.27×10^{-310} / 0	GWAS lead SNP		--	--	0.23	0.77
GSDMA	1.42×10^{-12} / 0	rs8065244	3.27×10^{-310} / 0	0.16	0.00	1.00	<0.001
PSMD3	1.20×10^{-7} / 3.7×10^{-4}	rs2302778	7.46×10^{-67} / 0	0.30	0.02	1.00	<0.001
MED24	7.10×10^{-42} / 0	rs11078934	7.84×10^{-85} / 0	0.92	0.16	0.80	0.20

FDR: false-discovery rate

[a] Selection criteria: genes in a window ±250 kB around the GWAS top-hit, FDR <0.05 in the association with expression

[b] Numerical underflow appears with values <3.27×10^{-310} and therefore, in the eQTL Consortium, all p-values below were set to this threshold. This might impact the calculations of the posterior probabilities of H3 and H4.

[c] LD taken from LDlink (https://ldlink.nih.gov/) using all European populations

H3: both mtDNA-CN and expression are associated with SNPs in the gene region, but with different causal variants

H4: both mtDNA-CN and expression are associated and share a single causal variant

Table S5: List of autosomal variants with p-values below 1x10^{-7} in either men or women from the sex-stratified meta-analysis.

Chr	Position	Lead SNP	A1	A2		Effect	StdErr	N	P-value	Freq A1 [a]	Freq A1 (1000G) [b]	Nearest Gene	P-value difference[c]	Distance BP	Adjustment
9	139617211	rs186793011	a	g	M	-0.154	0.127	5133	0.2263	0.006	0.003	*SNORA43/ DIPK1P*	1.76 x10^{-06}	3345	Age, sex, 4 PCs, erythrocyte, leukocyte and platelet count
					F	0.701	0.128	6211	4.21x10^{-8}	0.0048					

[a] Freq A1 = weighted average of frequency for allele 1 across all studies based on our meta-analysis results

[b] Freq A1 (1000G) = frequency of the A1 allele based on 1000 Genome Europeans

[c] Based on z-test of difference [22]: $Z_{diff} = \frac{(\beta_m - \beta_w)}{\sqrt{se_m^2 + se_w^2 - 2 \cdot r \cdot se_m \cdot se_w}}$

Abbreviations: Chr = Chromsome, BP = base pairs, PCs = principle components, M = men, F = women

Table S6: Results for lead SNPs, stratified for smoking categories adjusted for age, sex and 4PCs.

	**Never smoker (n=8,038)**			**Former smoker (n=5,514)**			**Current smoker (n=2,500)**			**p-value for difference**		
**Lead SNP**	Effect	StdErr	p-value	Effect	StdErr	p-value	Effect	StdErr	p-value	Never versus Former	Never versus Current	Former versus Current
**rs4895440**	-0.0744	0.0162	4.54×10^{-06}	-0.0793	0.0193	4.104×10^{-05}	-0.0711	0.0271	0.0086	0.8451	0.9165	0.8045
**rs56030650**	-0.0532	0.0145	0.0002	-0.0583	0.0173	0.0007	-0.0423	0.0236	0.0739	0.8204	0.6931	0.5830

Table S7: Mediation effects of SNPs rs56030650, respectively rs48955440 on mtDNA-CN in the AugUR (a) and CHRIS study (b).

a) AugUR study

		Effect (95% bootstrap CI)		% mediated (95% bootstrap CI)	
**Effect of rs56030650 on mtDNA-CN, adjusted for age, sex, RBC, PLT, smoking, 4 PCs**					
Indirect effect	WBC	-0.23 (-0.71, 0.17)		6.6 (-7.2, 25.8)	
Direct effect		-3.21 (-5.34, -0.62)		93.4 (74.2, 100)	
Total effect		-3.44 (-5.65, -0.84)			
**Effect of rs48955440 on mtDNA-CN, adjusted for age, sex, smoking, 4 PCs**					
	WBC		0.14 (-0.18, 0.55)		2.8 (-5.0, 12.3)
Indirect effect	RBC	2.02 (1.12, 2.86)	0.4 (-0.06, 0.68)	40.5 (15.8, 66.3)	8.0 (-3.4, 16.9)
	PLT		1.48 (0.71, 2.28)		29.7 (11.6, 49.4)
Direct effect		2.97 (0.56, 5.74)		59.5 (33.7, 84.2)	
Total effect		5.0 (2.48, 7.80)			

b) CHRIS study

		Effect (95% CI)		% mediated (95% CI)	
**Effect of rs56030650 on mtDNA-CN, adjusted for age, sex, RBC, PLT, smoking, 4 PCs**					
Indirect effect	WBC	-1.28 (-1.81, -0.81)		49.6 (21.5, 83.1)	
Direct effect		-1.31 (-2.61, -0.08)		50.4 (16.8, 78.7)	
Total effect		-2.60 (-4.02, -1.28)			
**Effect of rs48955440 on mtDNA-CN, adjusted for age, sex, smoking, 4 PCs**					
	WBC		1.35 (0.79, 1.88)		30.2 (12.9, 48.3)
Indirect effect	RBC	2.87 (2.27, 3.55)	0.48 (0.27, 0.76)	64.2 (39.4, 93.7)	10.7 (4.8, 18.7)
	PLT		1.04 (0.56, 1.56)		23.3 (10.1, 38.4)
Direct effect		1.61 (-0.11, 3.33)		36.0 (6.3, 54.5)	
Total effect		4.47 (2.74, 6.30)			

References

1 Binder, J. X. *et al.* COMPARTMENTS: unification and visualization of protein subcellular localization evidence. *Database (Oxford)* **2014**, bau012, doi:10.1093/database/bau012 (2014).

2 Boughton, A. P. *et al.* LocusZoom.js: Interactive and embeddable visualization of genetic association study results. *Bioinformatics*, doi:10.1093/bioinformatics/btab186 (2021).

3 Võsa, U. *et al.* Large-scale cis- and trans-eQTL analyses identify thousands of genetic loci and polygenic scores that regulate blood gene expression. *Nat Genet* **53**, 1300-1310, doi:10.1038/s41588-021-00913-z (2021).

4 Mungall, C. J. *et al.* The Monarch Initiative: an integrative data and analytic platform connecting phenotypes to genotypes across species. *Nucleic Acids Res* **45**, D712-D722, doi:10.1093/nar/gkw1128 (2017).

5 McInerney, T. W. *et al.* A globally diverse reference alignment and panel for imputation of mitochondrial DNA variants. *BMC Bioinformatics* **22**, 417, doi:10.1186/s12859-021-04337-8 (2021).

6 Weissensteiner, H. *et al.* HaploGrep 2: mitochondrial haplogroup classification in the era of high-throughput sequencing. *Nucleic Acids Res* **44**, W58-63, doi:10.1093/nar/gkw233 (2016).

7 Franz, M. *et al.* GeneMANIA update 2018. *Nucleic Acids Res* **46**, W60-W64, doi:10.1093/nar/gky311 (2018).

8 Curran, J. E. *et al.* Genetic determinants of mitochondrial content. *Hum Mol Genet* **16**, 1504-1514, doi:10.1093/hmg/ddm101 (2007).

9 López, S. *et al.* A genome-wide association study in the genetic analysis of idiopathic thrombophilia project suggests sex-specific regulation of mitochondrial DNA levels. *Mitochondrion* **18**, 34-40, doi:10.1016/j.mito.2014.09.004 (2014).

10 Cai, N. *et al.* Genetic Control over mtDNA and Its Relationship to Major Depressive Disorder. *Curr Biol* **25**, 3170-3177, doi:10.1016/j.cub.2015.10.065 (2015).

11 Workalemahu, T. *et al.* Genetic variations related to maternal whole blood mitochondrial DNA copy number: a genome-wide and candidate gene study. *J Matern Fetal Neonatal Med* **30**, 2433-2439, doi:10.1080/14767058.2016.1252747 (2017).

12 Li, Z. *et al.* Genetic variants in nuclear DNA along with environmental factors modify mitochondrial DNA copy number: a population-based exome-wide association study. *BMC Genomics* **19**, 752, doi:10.1186/s12864-018-5142-7 (2018).

13 Guyatt, A. L. *et al.* A genome-wide association study of mitochondrial DNA copy number in two population-based cohorts. *Hum Genomics* **13**, 6, doi:10.1186/s40246-018-0190-2 (2019).

14 Ganel, L. *et al.* Mitochondrial genome copy number measured by DNA sequencing in human blood is strongly associated with metabolic traits via cell-type composition differences. *Hum Genomics* **15**, 34, doi:10.1186/s40246-021-00335-2 (2021).

15 Hägg, S., Jylhävä, J., Wang, Y., Czene, K. & Grassmann, F. Deciphering the genetic and epidemiological landscape of mitochondrial DNA abundance. *Hum Genet* **140**, 849-861, doi:10.1007/s00439-020-02249-w (2021).

16 Gentiluomo, M. *et al.* Genome-wide association study of mitochondrial copy number. *Hum Mol Genet*, doi:10.1093/hmg/ddab341 (2021).

17 Longchamps, R. J. *et al.* Genome-wide analysis of mitochondrial DNA copy number reveals loci implicated in nucleotide metabolism, platelet activation, and megakaryocyte proliferation. *Hum Genet*, doi:10.1007/s00439-021-02394-w (2021).

18 Chong, M. *et al.* GWAS and ExWAS of blood Mitochondrial DNA copy number identifies 71 loci and highlights a potential causal role in dementia. *Elife* **11**, doi:10.7554/eLife.70382 (2022).

19 Pillalamarri, V. *et al.* Whole-exome sequencing in 415,422 individuals identifies rare variants associated with mitochondrial DNA copy number. *HGG Adv* **4**, 100147, doi:10.1016/j.xhgg.2022.100147 (2023).

20 Zaidi, A. A. *et al.* The genetic and phenotypic correlates of mtDNA copy number in a multi-ancestry cohort. *HGG Adv* **4**, 100202, doi:10.1016/j.xhgg.2023.100202 (2023).

21 Gupta, R. *et al.* Nuclear genetic control of mtDNA copy number and heteroplasmy in humans. *Nature*, doi:10.1038/s41586-023-06426-5 (2023).

22 Winkler, T. W. *et al.* Approaches to detect genetic effects that differ between two strata in genome-wide meta-analyses: Recommendations based on a systematic evaluation. *PLoS One* **12**, e0181038, doi:10.1371/journal.pone.0181038 (2017).
